# Sex-Specific Associations of Radiographic Knee Osteoarthritis and Pain with Distal Tibia Bone Microarchitecture: the Study of Muscle, Mobility and Aging (SOMMA)

**DOI:** 10.1007/s00223-026-01531-9

**Published:** 2026-04-27

**Authors:** Tong Yu, Andy Kin On Wong, Nina Z. Heilmann, Kerri S. Freeland, Bradley C. Nindl, Kristen J. Koltun, Andrew J. Burghardt, Julie M. Hughes, Peggy M. Cawthon, Jane A. Cauley, Elsa S. Strotmeyer, Nancy E. Lane

**Affiliations:** 1 Division of Epidemiology, Dalla Lana School of Public Health, University of Toronto, Toronto, ON Canada; 2 Schroeder Arthritis Institute, University Health Network, Toronto, ON Canada; 3 Joint Department of Medical Imaging, University Health Network, Toronto, ON Canada; 4 Department of Epidemiology, School of Public Health, University of Pittsburgh, Pittsburgh, PA USA; 5 Department of Internal Medicine, Wake Forest University School of Medicine, Winston-Salem, NC USA; 6 Neuromuscular Research Laboratory/Warrior Human Performance Research Center, Department of Sports Medicine and Nutrition, University of Pittsburgh, Pittsburgh, PA USA; 7 Department of Radiology and Biomedical Imaging, University of California, San Francisco, CA USA; 8 Military Performance Division, U.S. Army Research Institute of Environmental Medicine, Natick, MA USA; 9 Department of Epidemiology and Biostatistics, University of California, San Francisco, CA USA; 10 California Pacific Medical Center, Research Institute, San Francisco, CA USA; 11 Division of Rheumatology and Clinical Allergy, Department of Medicine, U.C. Davis Health, Sacramento, CA USA

**Keywords:** Knee osteoarthritis, HR-pQCT, Knee pain, Sex differences, Older adults

## Abstract

Knee osteoarthritis (OA) is the most common joint disease and despite being associated with higher areal bone mineral density (aBMD), increases fracture risk. This paradox suggests that in addition to mobility and fall concerns, microarchitectural deficits, not captured by DXA, may contribute to skeletal fragility in knee OA. This study aimed to examine cross-sectional associations of radiographic knee OA and knee pain with bone microarchitecture at the distal tibia and distal radius, assessed by high-resolution peripheral quantitative computed tomography (HR-pQCT), in older adults. We studied 107 men and 159 women (age 77 ± 5 years) from the study of muscle, mobility and aging (SOMMA) who had knee radiographs and HR-pQCT scans. Radiographic knee OA was defined as Kellgren–Lawrence grade (KLG) 3–4, and knee pain was assessed using the Brief Pain Inventory questionnaire. Linear regression models estimated sex-specific associations of knee OA and pain with standardized HR-pQCT parameters, adjusting for age, BMI, FN aBMD, physical activity, fracture history, smoking, alcohol, and medication use. In women, at the distal tibia, radiographic knee OA was associated with lower cortical vBMD (B = − 0.55 SD, 95% CI − 0.91, − 0.20). In men, knee pain was associated with lower total and cortical vBMD, thinner cortices, and larger trabecular area. No associations were detected at the distal radius. Radiographic knee OA was more strongly associated with lower distal tibia cortical density in women, while pain was associated with overall structural and density deficits at distal tibia in men. These sex-specific patterns may inform fracture risk and OA etiology.

**Supplementary Information:**

The online version contains supplementary material available at 10.1007/s00223-026-01531-9.

## Introduction

Knee osteoarthritis (OA) is the most prevalent joint disease and a leading cause of disability, affecting nearly one in five adults ≥ 45 years and up to 37% of adults ≥ 60 years in the United States [1, 2]. With an aging population and rising obesity rates, the prevalence and burden of knee OA are projected to increase from 13.8 to 15.7% by 2032 [3]. Beyond pain and disability, knee OA has been associated with an elevated risk of fractures [4–6]. Several mechanisms may underlie this association of knee OA and fracture, including reduced mobility, impaired balance, increased fall risk, and changes in bone health [6–8].

Bone health may serve as an important intermediary in the relationship between knee OA and fracture. Locally, knee OA is characterized by subchondral bone remodeling and structural changes, including sclerosis, trabecular alterations, and increased turnover, that contribute to disease progression and pain [7, 9]. Systemically, skeletal integrity is often assessed using areal bone mineral density (aBMD), a well-established predictor of fracture risk. However, prior studies have reported that individuals with knee OA have higher aBMD compared to those without OA [10–14], yet paradoxically still experience a higher risk of fractures [4–6]. This discrepancy suggests that aBMD, as measured by the two-dimensional modality—Dual-energy X-ray Absorptiometry (DXA), may not fully capture aspects of bone health most relevant to fracture risk in knee OA, and that microarchitectural parameters may provide additional insights.

High-resolution peripheral quantitative computed tomography (HR-pQCT) has advanced the understanding of how bone microarchitecture contributes to fracture risk in osteoporosis [15]. Although pQCT and HR-pQCT have been applied to study bone structure at the knee [16–18], technical constraints, such as gantry size and joint motion, have limited investigations to younger patients with post-traumatic OA or non-overweight individuals [19, 20]. These studies reported inconsistent findings: no clear association between subchondral bone volumetric BMD (vBMD) and radiographic knee OA by Kellgren–Lawrence grade [17, 21], but the presence of an association between lower proximal tibia trabecular vBMD and more severe symptoms in a small sample [22]. Importantly, these analyses were limited by minimal adjustment for OA-related risk factors, typically only age and BMI.

HR-pQCT imaging of the distal tibia (a weight-bearing site) and distal radius (a non-weight-bearing site) provides measures of cortical and trabecular structure, both of which are strongly linked to fracture risk [15]. Yet, to date, no studies have examined distal tibia bone microstructure in relation to knee OA or knee pain. Investigating these associations could provide novel insight into systemic skeletal pathways that connect knee OA with fracture susceptibility.

Therefore, in this study, we aimed to evaluate the cross-sectional associations among both radiographic knee OA and knee pain with HR-pQCT-derived bone microarchitecture parameters at the distal tibia and distal radius, independent of aBMD and other potential confounders, among older adults. We hypothesized that individuals with knee OA or knee pain would have lower vBMD and lower failure load at distal tibia, but not at distal radius, compared to individuals without knee OA or without knee pain.

## Methods

### Study Population

#### SOMMA Study Inclusion/Exclusion

The Study of Muscle, Mobility and Aging (SOMMA) aims to determine the biological processes that contribute to changes in mobility and fitness with aging [23, 24]. SOMMA recruited participants at two clinical sites (University of Pittsburgh and Wake Forest University School of Medicine). Study participants were included if they were ≥ 70 years old, able to walk ≥ 0.6 m/s (4-m test), complete a 400-m walk, free of life-threatening diseases, and had no contraindications to magnetic resonance imaging or to muscle tissue collection. Individuals were excluded from the study if they reported inability to walk a ¼ mile (400 m) or climb a flight of stairs, walked slower than 0.6 m/s on a 4-m walk, had BMI > 40 kg/m^2^, had active cancer or advanced chronic diseases, such as heart failure, renal failure on dialysis, Parkinson’s disease or dementia [23].

**Fig. 1:**
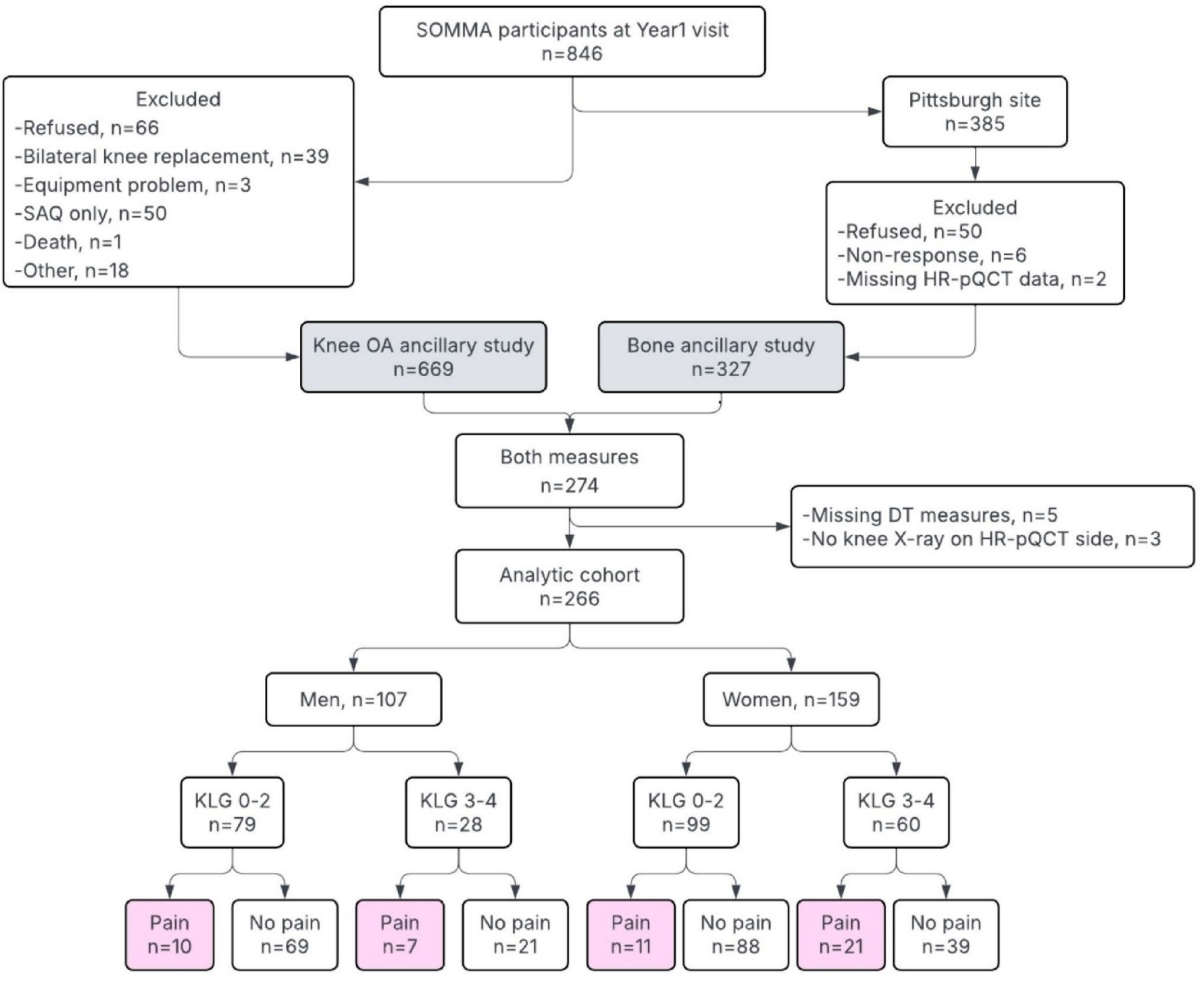
Study flow diagram. ^a^Kellgren–Lawrence grades (KLG) as based on worse KLG between tibio-femoral joint and patello-femoral joint on the same side of HR-pQCT scan. ^b^Pain was assessed by brief pain inventory

Two SOMMA ancillary studies additionally expanded data collection beyond the core protocol to capture detailed bone and joint measures. The SOMMA Knee OA ancillary study invited SOMMA participants to return for the first annual follow-up visit between June 2020 and January 2023, which included a standing bilateral knee radiograph (n = 669, Fig. 1 Study flow diagram). The SOMMA Bone ancillary study enrolled SOMMA participants who attended the first annual follow-up visit at the University of Pittsburgh site and obtained an HR-pQCT scan of the distal tibia and radius (n = 327).

#### Present Study Inclusion/Exclusion

In the current study, we included participants who were simultaneously enrolled in the SOMMA Knee OA and SOMMA Bone ancillary studies (Pittsburgh site only) (Fig. 1). All participants provided written informed consent, and the study was approved by the Western Institutional Review Board (WCGIRB #20180764) for all participating sites [23, 25].

### Outcome: HR-pQCT Bone Parameters

HR-pQCT scans were acquired at the first annual visit using Scanco XtremeCT II (Scanco Medical AG, Brüttisellen, Switzerland) with a nominal isotropic voxel size of 61 μm. Scanning was performed on the right leg and ipsilateral forearm except in the case of contraindications (i.e., prior fractures, metal artifacts, significant weaknesses, or recent unloading for ≥ 6 weeks for the tibial site), in which case the contralateral side was scanned. Trained operators acquired scans of the distal radius (fixed offset method at 9.5 mm from the distal endplate) and distal tibia (fixed offset method at 22.5 mm from the tibial plafond) [26]. Representative scout views illustrating the scanned regions of interest are shown in Supplementary Fig. 1.

Machines were calibrated daily using a Scanco XtremeCT II phantom to monitor for drift beyond ± 8 mg HA/cm^3^. A central observer read and excluded images with motion grades of 4 or 5 using a previously reported method [27, 28]. This method was validated against the first-generation XtremeCT [29] and has been applied in previous studies [30]. At the distal radius and distal tibia, vBMD (mg HA/cm^3^) and cross-sectional area (mm^2^) of the total (Tt.), cortical (Ct.) and trabecular (Tb.) bone were measured. Cortical and trabecular thickness (Ct.Th, Tb.Th, mm), and trabecular number (Tb.N, mm^−1^) were calculated directly. Failure load (N) when 2% of the elements exceeded 0.7% strain [31] was estimated using linear elastic micro-finite element analysis (μFEA). All HR-pQCT parameters were standardized for each sex. HR-pQCT bone parameters including Tt., Ct., Tb.vBMD and μFEA failure load at the distal tibia, a weight-bearing site, were the primary outcomes, while other parameters at the distal tibia and distal radius were secondary outcomes.

### Exposure: Radiographic Knee OA

Bilateral, fixed-flexion, weight-bearing posteroanterior radiographs of the knees were obtained at year 1 following well-validated methods [32]. The severity of radiographic knee OA at tibio-femoral (TF) and patello-femoral (PF) joints were separately determined with Kellgren–Lawrence grades (KLG) (scale 0–4) [33]. The inter-reader reliability for KLG scoring was good with a kappa statistic of 0.75 [34]. Consensus readings were also performed on knees with unusual combinations of KLG, joint space width, and osteophytes [34]. Participants were categorized into “no/mild” radiographic knee OA based on KLG (0–2) of the worse joint (TF or PF) on the same side of the HR-pQCT scan; otherwise, they were categorized into “moderate/severe” radiographic knee OA (KLG 3–4).

### Exposure: Knee Pain

General knee pain and functional knee pain were assessed by a series of semi-quantitative pain questionnaires at the Year 1 follow-up visit. Presence of general knee pain was assessed using the Brief Pain Inventory (BPI) as guided by a homunculus. Participants were categorized into having BPI knee pain if they reported current painful knee on the same side as the HR-pQCT scans. Functional knee pain was defined by the presence of monthly or more frequent knee pain (mild or beyond vs. none) while going up or down the stairs, and while walking on a flat surface, using the question “What amount of knee pain have you experienced in the last week during a. going up or down the stairs, b. walking on flat surface (0: None, 1: Mild, 2: Moderate, 3: Severe, 4: Extreme)”.

### Other Covariates

Age (continuous), race (White vs. non-White), education (college graduate or more vs. some college or less), annual household income (≥ $50,000 vs. < $50,000), prevalent fracture(s) since age 50 (yes vs. no) at baseline, smoking status (ever smoker vs. never smoker), and alcohol use (non-drinker, 0–2 drinks, and ≥ 3 drinks per week) were collected using standardized questionnaires at baseline. Standing height (mm) was measured using a stadiometer, weight was measured using a digit scale (kg), and BMI was calculated as weight (kg)/[height(m)]^2^. Femoral neck (FN) aBMD was measured using Dual-energy X-ray absorptiometry (DXA, Lunar iDXA, GE Healthcare, Illinois, US). Multimorbidity index was calculated as the number of comorbidities from 0 to 11, including (1) non-skin cancer, (2) cardiac arrhythmia, (3) chronic kidney disease, (4) chronic obstructive pulmonary disease, (5) coronary artery disease, (6) congestive heart failure, (7) dementia, (8) depression, (9) diabetes, (10) stroke, and (11) aortic stenosis. Use of osteoporosis medications (yes vs. no, including bisphosphonates, calcitonin, raloxifene, denosumab, romosozumab, teriparatide, or abaloparatide) and pain medications (yes vs. no, including oral corticosteroid, COX-II inhibitor, opioid analgesics, and prescription NSAIDs) within the past year was collected from the medication inventory at the first annual visit. Physical activity (mean steps per day) was assessed at the baseline visit using an activPAL4™ micro accelerometer by PAL Technologies Ltd (Glasgow, Scotland). Participants were asked to wear the activPAL along the midline of the right thigh for 7 consecutive 24-h periods.

### Statistical Analyses

The analyses were conducted at the participant level. The study sample for this analysis included individuals with complete data including at a minimum, KLG and pain data from the same side as the distal tibia HR-pQCT scan. We compared baseline characteristics of study participants with moderate/severe radiographic knee OA (KLG 3–4) to those with no/mild radiographic knee OA (KLG 0–2) overall and by sex. Continuous variables were compared using Student’s t-test or Kruskal–Wallis tests if the distributions were skewed. Categorical variables were compared using a Chi-square test or Fisher’s exact test in the case of small cell sizes (≤ 5). We made similar comparisons by BPI knee pain (yes vs. no).

To assess cross-sectional associations, we first plotted the unadjusted mean of standardized HR-pQCT parameter patterns across the full KLG (0–4) spectrum. We then used multivariable linear regression models to estimate average differences (B: regression coefficients) in standardized HR-pQCT parameters comparing dichotomous radiographic knee OA (KLG 3–4 vs. 0–2) and dichotomous knee pain (yes vs. no) and corresponding 95% confidence intervals (95% CI). Model adjustments were guided by a priori risk factors for knee OA and bone structure, including age, BMI, prevalent fracture since age 50, smoking, alcohol use, physical activity, and FN aBMD T-score. We additionally adjusted for osteoporosis medication use for radiographic knee OA models, and pain medication use for knee pain models. All models were performed separately by biological sex. Sex differences in regression coefficients were tested using sex-by-comparator interaction terms.

We considered several sensitivity analyses. For radiographic knee OA outcomes, we assessed the contrast between “no” (KLG 0–1) and “mild-to-severe” (KLG 2–4) radiographic knee OA. In addition, we assessed radiographic knee OA at TF and PF joints separately to determine if bone parameters had distinct associations with radiographic knee OA at each knee joint. The same set of confounders were used in this sensitivity analysis. For knee pain outcomes, we assessed persistent pain defined as having general or functional knee pain at both baseline and Year 1 visit, representing persistent knee pain over 1 year before the HR-pQCT scan. Finally, we also used a more stringent definition of knee OA—simultaneous radiographic knee OA (KLG 3–4 at TF or PF joint) and BPI knee pain as the exposure to determine if distal site bone parameters were associated with both diagnostic criteria.

A two-sided significance level of 0.05 was used. We additionally present significant *p* values for all secondary outcomes after accounting for multiple comparisons using the Benjamini–Hochberg correction. All analyses were performed using R 4.2.2 (R Core Team).

## Results

### Cohort and Participant Characteristics

From the 669 participants in the SOMMA Knee OA ancillary study and 327 participants in the SOMMA bone ancillary study, 266 participants (107 men and 159 women) were included in the present analyses. Mean age was 77.2 years (SD 4.7), mean BMI was 27.7 kg/m^2^ (SD 4.6), and 87% self-reported being of White race. Men and women with moderate/severe radiographic knee OA (KLG 3–4) had significantly lower physical activity than the no/mild group. Women with moderate/severe radiographic knee OA also had higher BMI, lower annual household income, and reported lower frequency of ever smoking, drinking, or use of osteoporosis medications compared to the no/mild group (Table 1). Men and women with BPI knee pain had lower physical activity and higher BMI, and women with BPI knee pain were more likely to have a fracture history before age 50 (Supplementary Table 1).

**Table 1:** Participant characteristics in SOMMA *Knee OA* and *Bone* ancillary studies by sex and radiographic knee OA (as determined by KLG, with levels 3–4 indicative of advanced disease)

	Men			Women		
	KLG 0–2	KLG 3–4	*p* value	KLG 0–2	KLG 3–4	*p* value
	*N* = 79	*N* = 28		*N* = 99	*N* = 60	
Age (years)	77.2 (4.4)	77.1 (4.5)	0.944	**76.6 (4.3)**	**78.3 (5.5)**	**0.045**
White race	71 (89.9%)	28 (100.0%)	0.108	83 (83.8%)	48 (80.0%)	0.688
BMI (kg/m^2^)	27.4 (4.3)	28.2 (3.8)	0.379	**26.9 (5.0)**	**29.1 (4.3)**	**0.005**
Weight, kg	81.6 (13.5)	86.1 (14.1)	0.147	**68.5 (12.6)**	**73.9 (12.0)**	**0.009**
Height, m	1.7 (0.1)	1.7 (0.1)	0.199	1.6 (0.1)	1.6 (0.1)	0.724
Income < $50,000	21 (27.6%)	7 (28.0%)		**40 (43.5%)**	**34 (69.4%)**	**0.006**
Fracture since age 50	8 (10.1%)	6 (21.4%)	0.189	28 (28.6%)	19 (31.7%)	0.815
Ever smoker	35 (44.3%)	15 (53.6%)	0.533	**52 (53.1%)**	**19 (31.7%)**	**0.014**
Alcohol drink per week			0.901			**0.002**
No drinks	25 (31.6%)	9 (32.1%)		41 (42.3%)	39 (67.2%)	
0–2 drink	26 (32.9%)	8 (28.6%)		24 (24.7%)	13 (22.4%)	
≥ 3 drinks	28 (35.4%)	11 (39.3%)		32 (33.0%)	6 (10.3%)	
Physical activity (steps/day)	**7287.0 (3631.9)**	**5700.8 (2165.1)**	**0.018**	**7212.0 (3230.8)**	**5688.9 (2926.6)**	**0.006**
Multi-comorbidity index	1.0 [0.0;1.0]	1.0 [0.0;1.0]	0.398	0.0 [0.0;1.0]	1.0 [0.0;1.0]	0.341
Osteoporosis medication	1 (1.3%)	0 (0.0%)	1.000	**18 (18.2%)**	**2 (3.3%)**	**0.013**
Prescription pain medication	3 (3.8%)	4 (14.3%)	0.075	15 (15.2%)	5 (8.3%)	0.312
Femoral neck BMD (g/cm^2^)	0.9 (0.1)	0.9 (0.2)	0.862	0.8 (0.1)	0.8 (0.1)	0.220
Tt. knee pain score (0–13)	**1.5 (2.0)**	**2.7 (2.6)**	**0.027**	**1.6 (2.2)**	**3.1 (3.0)**	**0.001**
Bone parameters at DT						
Tt. vBMD (mg HA/cm^3^)	309.6 (46.5)	292.2 (56.6)	0.150	241.3 (39.8)	246.2 (57.4)	0.559
Tl. area (mm^2^)	861.2 (142.2)	913.1 (202.5)	0.219	697.6 (108.5)	674.5 (134.0)	0.261
FEA failure load (N)	12,868.9 (2144.4)	12,361.5 (2820.0)	0.391	7938.7 (1452.0)	7927.5 (1956.6)	0.969
Ct. vBMD (mg HA/cm^3^)	**828.1 (68.7)**	**793.8 (69.4)**	**0.029**	775.2 (69.5)	748.7 (92.1)	0.057
Ct. area (mm^2^)	157.6 (25.8)	149.2 (28.4)	0.178	101.0 (19.2)	100.9 (27.0)	0.980
Ct. thickness (mm)	1.6 (0.3)	1.5 (0.3)	0.190	1.2 (0.2)	1.2 (0.3)	0.671
Tb. vBMD (mg HA/cm^3^)	194.1 (31.3)	189.9 (40.1)	0.622	150.1 (35.5)	156.9 (48.9)	0.350
Tb. area (mm^2^)	728.9 (130.5)	770.1 (207.2)	0.332	602.0 (111.6)	597.8 (111.7)	0.818
Tb. thickness (mm)	0.3 (< 0.1)	0.3 (< 0.1)	0.862	0.3 (< 0.1)	0.3 (< 0.1)	0.238
Tb. number (mm^−1^)	1.5 (0.2)	1.5 (0.3)	0.689	1.3 (0.2)	1.3 (0.3)	0.414

*KLG* Kellgren Lawrence grade, *Tt.* total, *Ct.* cortical, *Tb.* trabecular, *FEA* finite element analysis, *Th* thickness, *N* number, *CI* confidence interval

Bold text: statistically significant (*p* < 0.05)

### Unadjusted HR-pQCT Parameters by KLG

**Fig. 2:**
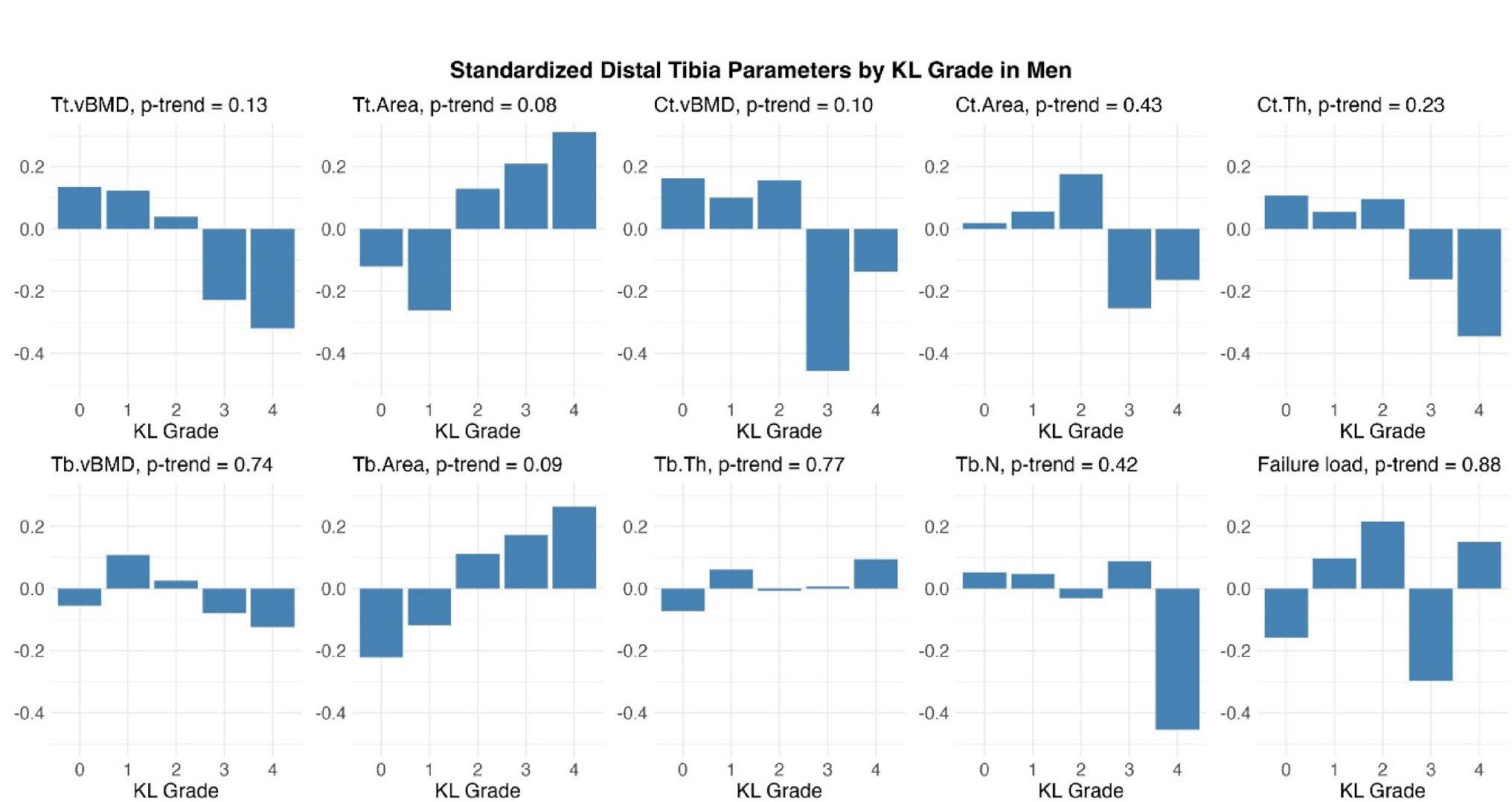
Standardized distal tibia HR-pQCT parameters by Kellgren–Lawrence grade in men

**Fig. 3:**
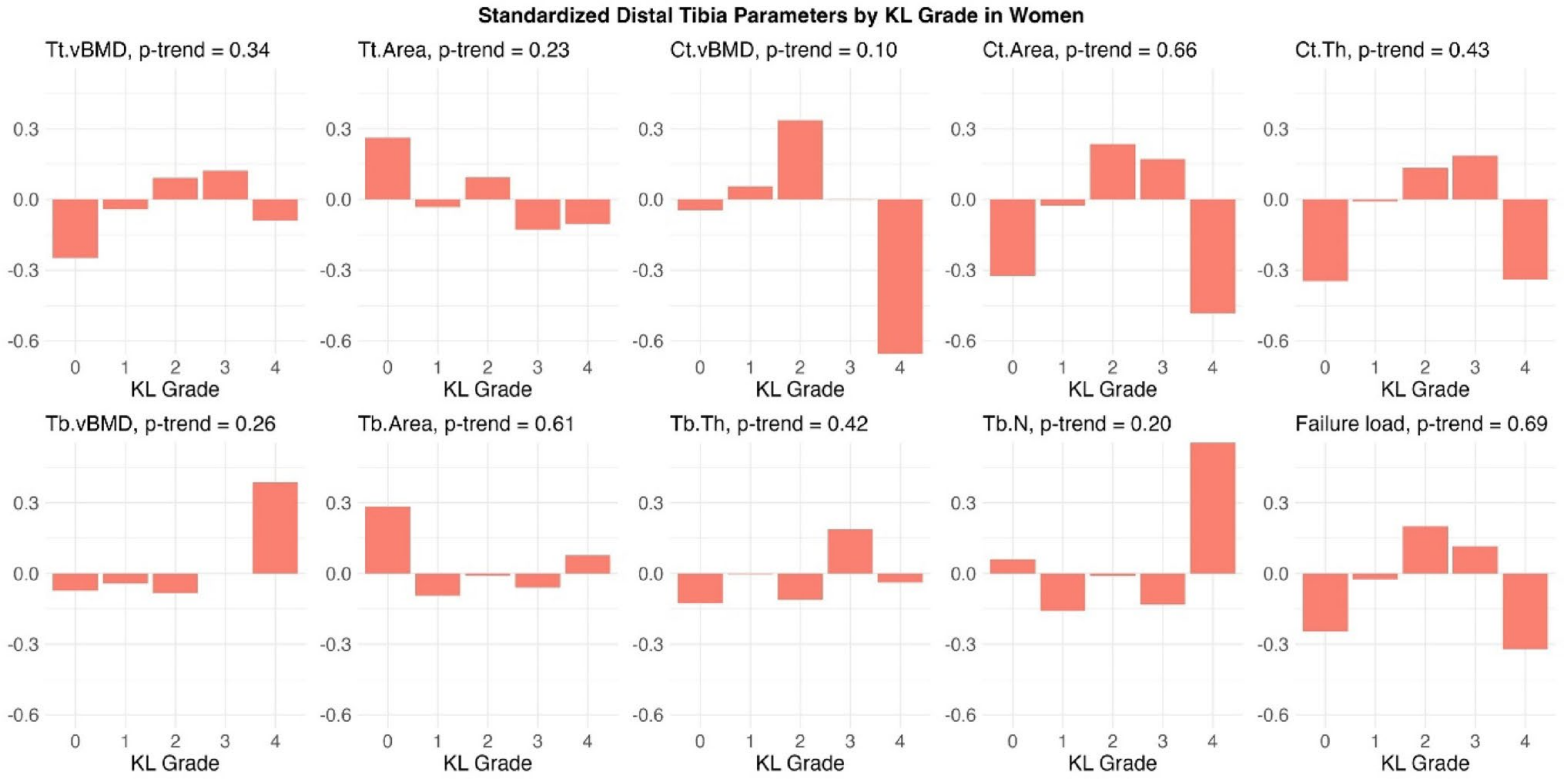
Standardized distal tibia HR-pQCT parameters by Kellgren–Lawrence grade in Women

In the unadjusted bar plots of sex-specific standardized distal tibia HR-pQCT parameters in men, higher KLG was correlated with lower Ct.vBMD, higher Tt.Area and higher Tb.Area with a marginally significant p-value for linear trend (*p* ≤ 0.10; Fig. 2 Standardized distal tibia HR-pQCT parameters by Kellgren–Lawrence grade in men). In women, a lower Ct.vBMD was primarily observed in individuals with moderate/severe knee OA. A few parameters also showed bi-modal distributions, where both women without radiographic knee OA (KLG 0) and those with severe disease (KLG 4) had smaller Ct.Area, lower Ct.Th and lower failure load (Fig. 3 Standardized distal tibia HR-pQCT parameters by Kellgren–Lawrence grade in women).

### HR-pQCT Parameters and Radiographic Knee OA

In multivariable-adjusted linear regression models, only in women, the presence of moderate/severe radiographic knee OA was associated with 0.55 standard deviation (SD) (95% CI 0.20–0.91) lower Ct.vBMD at the distal tibia, compared to those with no/mild radiographic knee OA. In men, radiographic knee OA was associated with 0.45 SD lower Ct.vBMD (*p* = 0.090) but was not statistically significant (Table 2). At the distal radius—a non-weight-bearing site—associations were in the same directions as the distal tibia in women. However, none of the HR-pQCT parameters were associated with radiographic knee OA at the 0.05 level (Supplementary Table 2a).

**Table 2:** Associations between standardized HR-pQCT parameters at the distal tibia (DT) and radiographic knee OA (as determined by KLG, with levels 3–4 indicative of advanced disease) in the SOMMA *Knee OA* and *Bone* ancillary studies as determined from multivariable linear regression models

DT HR-pQCT	Radiographic knee OA: KLG 3–4 vs. 0–2					
	Men (N = 82)			Women (N = 121)		
	B	95% CI	*p* value	B	95% CI	*p* value
Tt. vBMD	− 0.32	(− 0.81, 0.18)	0.218	− 0.10	(− 0.46, 0.26)	0.575
Tt. Area^#^	0.46	(− 0.08, 1.00)	0.102	− 0.26	(− 0.66, 0.14)	0.203
Ct. vBMD	− 0.45	(− 0.96, 0.06)	0.090	**− 0.55**	**(− 0.91, − 0.20)**	0.003*
Ct. Area	− 0.30	(− 0.76, 0.16)	0.207	− 0.26	(− 0.61, 0.09)	0.147
Ct. Th	− 0.35	(− 0.84, 0.15)	0.176	− 0.14	(− 0.51, 0.24)	0.480
Tb. vBMD	− 0.01	(− 0.46, 0.44)	0.967	0.06	(− 0.32, 0.44)	0.762
Tb. Area	0.35	(− 0.19, 0.89)	0.204	− 0.08	(− 0.49, 0.32)	0.684
Tb. Th	− 0.09	(− 0.58, 0.40)	0.727	0.06	(− 0.36, 0.48)	0.784
Tb. N	− 0.02	(− 0.55, 0.50)	0.933	0.08	(− 0.33, 0.49)	0.688
FEA failure load	− 0.13	(− 0.51, 0.26)	0.518	− 0.31	(− 0.62, 0.00)	0.054

*OA* osteoarthritis, *KLG* Kellgren Lawrence Grade, *Tt.* total, *Ct.* cortical, *Tb.* trabecular, *FEA* finite element analysis, *Th* thickness, *N* number, *CI* confidence interval

Model adjustments: age, BMI, fracture since age 50, smoking (ever vs. never), drinking (≥ 2, 0–2, vs. 0 drinks/week), physical activity (ActivPAL4™, steps/day), femoral neck BMD T-score, osteoporosis medication

^#^denotes *p* < 0.1 for interaction between HR-pQCT parameters and sex

Bold text: statistically significant (*p* < 0.05)

*denotes *p* < 0.05 after accounting for multiple comparison

### HR-pQCT Parameters and Knee Pain

In men, the presence of general knee pain assessed by BPI was associated with 0.93 SD lower Tt.vBMD (95% CI 0.36–1.49), 0.75 SD lower Ct.vBMD (95% CI 0.14–1.35), 0.74 SD lower Ct.Th (95% CI 0.15–1.32), and 0.88 SD larger Tb.Area (95% CI 0.26–1.49) at distal tibia, compared to those without general knee pain. In women, associations were not significant (Table 3).

**Table 3:** Associations between standardized HR-pQCT parameters at the distal tibia (DT) and general knee pain assessed by BPI in the SOMMA *Bone* and *Knee OA* ancillary studies as determined from multivariable linear regression models

DT HR-pQCT	General knee pain by BPI (yes vs. no)					
	Men (N = 83)			Women (N = 122)		
	B	95% CI	*p* value	B	95% CI	*p* value
Tt. vBMD^#^	**− 0.93**	**(− 1.49, − 0.36)**	0.002*	− 0.11	(− 0.52, 0.30)	0.604
Tt. Area	0.48	(− 0.16, 1.13)	0.144	0.11	(− 0.36, 0.57)	0.652
Ct. vBMD	**− 0.75**	**(− 1.35, − 0.14)**	0.018*	− 0.35	(− 0.78, 0.08)	0.109
Ct. Area	**− 0.61**	**(− 1.15, − 0.06)**	**0.033**	− 0.29	(− 0.69, 0.11)	0.161
Ct. Th	**− 0.74**	**(− 1.32, − 0.15)**	0.016*	− 0.25	(− 0.68, 0.18)	0.259
Tb. vBMD	− 0.44	(− 0.97, 0.10)	0.113	0.13	(− 0.31, 0.57)	0.577
Tb. Area	**0.88**	**(0.26, 1.49)**	0.006*	0.09	(− 0.38, 0.56)	0.701
Tb. Th	− 0.07	(− 0.67, 0.52)	0.809	− 0.09	(− 0.58, 0.40)	0.723
Tb. N	− 0.31	(− 0.94, 0.32)	0.342	0.34	(− 0.13, 0.81)	0.160
FEA failure load	− 0.31	(− 0.77, 0.15)	0.186	− 0.12	(− 0.48, 0.24)	0.523

*OA* osteoarthritis, *BPI* brief pain inventory, *Tt.* total, *Ct.* cortical, *Tb.* trabecular, *FEA* finite element analysis, *Th* thickness, *N* number, *CI* confidence interval

Model adjustments: age, BMI, fracture since age 50, smoking (ever vs. never), drinking (≥ 2, 0–2, vs. 0 drinks/week), physical activity (ActivPAL4™, steps/day), femoral neck BMD T-score, pain medication

^#^denotes *p* < 0.1 for interaction between HR-pQCT parameters and sex

Bold text: statistically significant (*p* < 0.05)

*denotes *p* < 0.05 after accounting for multiple comparison

Only in men, the presence of knee pain when climbing stairs was associated with 0.49 SD lower Ct.Area (95% CI 0.03–0.94) and 0.55 higher Tb.Th (95% CI 0.07–1.04) at distal tibia, compared to those without knee pain when climbing stairs (Table 4). Similar associations at the distal radius (Ct.Area and Tb.Th) were also observed in men (Supplementary Table 4a). Only in women, the presence of knee pain when walking on flat surfaces was associated with 0.46 SD higher Ct.Area (95% CI 0.01–0.91) and 0.58 SD higher Ct.Th (95% CI 0.10–1.06) at distal tibia, compared to those without knee pain when walking flat (Table 5). None of the HR-pQCT parameters at distal radius were significantly associated with the presence of general or functional knee pain when walking on flat surfaces (Supplementary Tables 3a and 5a).

**Table 4:** Associations between standardized HR-pQCT parameters at the distal tibia (DT) and functional knee pain when climbing stairs in the SOMMA *Bone* and *Knee OA* ancillary studies as determined from multivariable linear regression models

DT HR- pQCT	Knee pain when climbing stairs (yes vs. no)					
	Men (N = 83)			Women (N = 122)		
	B	95% CI	*p* value	B	95% CI	*p* value
Tt. vBMD	0.10	(− 0.40, 0.60)	0.704	0.07	(− 0.31, 0.44)	0.729
Tt. Area	− 0.33	(− 0.87, 0.21)	0.231	− 0.09	(− 0.51, 0.33)	0.682
Ct. vBMD	− 0.11	(− 0.63, 0.42)	0.694	0.32	(− 0.07, 0.71)	0.110
Ct. Area^#^	**− 0.49**	**(− 0.94, − 0.03)**	**0.039**	0.18	(− 0.18, 0.55)	0.329
Ct. Th	− 0.22	(− 0.73, 0.28)	0.394	0.16	(− 0.23, 0.55)	0.433
Tb. vBMD	0.35	(− 0.10, 0.79)	0.133	− 0.16	(− 0.56, 0.24)	0.437
Tb. Area	− 0.36	(− 0.89, 0.18)	0.196	− 0.15	(− 0.58, 0.27)	0.487
Tb. Th*	**0.55**	**(0.07, 1.04)**	**0.028**	− 0.19	(− 0.63, 0.25)	0.402
Tb. N	0.31	(− 0.22, 0.84)	0.252	0.14	(− 0.29, 0.58)	0.518
FEA failure load	− 0.31	(− 0.69, 0.07)	0.117	− 0.06	(− 0.39, 0.27)	0.715

*OA* osteoarthritis, *Tt.* total, *Ct.* cortical, *Tb.* trabecular, *FEA* finite element analysis, *Th* thickness, *N* number, *CI* confidence interval

Model adjustments: age, BMI, fracture since age 50, smoking (ever vs. never), drinking (≥ 2, 0–2, vs. 0 drinks/week), physical activity (ActivPAL4™, steps/day), femoral neck BMD T-score, pain medication

*denotes *p* < 0.05 for interaction between HR-pQCT parameters and sex; ^#^denotes *p* < 0.1 for interaction between HR-pQCT parameters and sex

Bold text: statistically significant (*p* < 0.05)

**Table 5:** Associations between standardized HR-pQCT parameters at the distal tibia (DT) and functional knee pain when walking on a flat surface in the SOMMA *Bone* and *Knee OA* ancillary studies as determined from multivariable linear regression models

DT HR-pQCT	knee pain when walking flat (yes vs. no)					
	Men (N = 83)			Women (N = 122)		
	B	95% CI	*p* value	B	95% CI	*p* value
Tt. vBMD	− 0.04	(− 0.68, 0.60)	0.902	0.32	(− 0.15, 0.79)	0.181
Tt. Area	− 0.22	(− 0.91, 0.46)	0.527	− 0.42	(− 0.94, 0.10)	0.118
Ct. vBMD	− 0.14	(− 0.81, 0.52)	0.674	0.40	(− 0.08, 0.89)	0.107
Ct. Area	− 0.18	(− 0.78, 0.41)	0.546	**0.46**	**(0.01, 0.91)**	**0.048**
Ct. Th	0.07	(− 0.58, 0.71)	0.843	**0.58**	**(0.10, 1.06)**	**0.019**
Tb. vBMD	− 0.03	(− 0.61, 0.54)	0.913	− 0.16	(− 0.66, 0.34)	0.528
Tb. Area	− 0.29	(− 0.97, 0.39)	0.405	− 0.50	(− 1.02, 0.03)	0.067
Tb. Th	0.53	(− 0.09, 1.16)	0.095	0.28	(− 0.27, 0.83)	0.323
Tb. N	− 0.41	(− 1.08, 0.25)	0.230	− 0.11	(− 0.65, 0.43)	0.702
FEA failure load	− 0.27	(− 0.75, 0.22)	0.280	0.07	(− 0.35, 0.48)	0.754

*OA* osteoarthritis, *Tt.* total, *Ct.* cortical, *Tb.* trabecular, *FEA* finite element analysis, *Th* thickness, *N* number, *CI* confidence interval

Model adjustments: age, BMI, fracture since age 50, smoking (ever vs. never), drinking (≥ 2, 0–2, vs. 0 drinks/week), physical activity (ActivPAL4™, steps/day), femoral neck BMD T-score, pain medication

Bold text: statistically significant (*p* < 0.05)

### Sensitivity Analyses: TF vs. PF Joints

In the sensitivity analyses, 19 out of 82 men and 22 out of 121 women had radiographic knee OA at the TF joint, 10 out of 82 men and 35 out of 121 women had radiographic knee OA at the PF joint. Only in women, the presence of radiographic knee OA at the PF joint was associated with 0.87 SD lower Ct.vBMD, 0.73 SD smaller Ct.Area, 0.62 SD lower Ct.Th, and 0.40 SD lower failure load at the distal tibia. Similar associations were also observed for parameters at the distal radius. The presence of radiographic knee OA at the TF joint was not significantly associated with any HR-pQCT parameters at distal tibia (Supplementary Tables 6a–7b).

### Sensitivity Analyses: Contrast Between No vs. Any Radiographic Knee OA

When comparing bone parameters between people with any radiographic knee OA (KLG 2–4) and people without knee OA at PF joint (KLG 0–1), contrary to the above results, only in men, the presence of OA was associated with higher Tt.vBMD, larger Tt.Area, larger Ct.Area, and lower Ct.Th (Supplementary Table 7b).

### Sensitivity Analyses: Persistent Knee Pain

When assessing persistent knee pain, only 5 men and 7 women reported persistent general knee pain, 13 men and 28 women reported persistent functional knee pain when climbing stairs, and 4 men and 14 women reported persistent functional knee pain when walking flat. We did not observe significant associations between persistent knee pain and bone parameters.

### Sensitivity Analyses: Simultaneous Radiographic Knee OA and BPI Knee Pain

At the distal tibia, 6 out of 82 men and 17 out of 121 women had simultaneous radiographic knee OA and BPI knee pain. Only in women, the presence of simultaneous conditions was associated with 0.67 SD lower Ct.vBMD and 0.62 SD higher Tb.N. At the distal radius, 5 out of 75 men and 16 out of 108 women had simultaneous radiographic knee OA and BPI knee pain. Only in men, the presence of simultaneous conditions was associated with 1.20 SD larger Ct.Area, 0.94 higher Ct.Th, and 1.07 higher FEA failure load (Supplementary Table 8a).

## Discussion

### Summary of Results

In this cross-sectional study of generally healthy older adults, we observed sex-specific associations between knee OA outcomes and bone microarchitecture at the distal tibia measured by HR-pQCT. In women, radiographic knee OA was associated with lower cortical vBMD after multivariable adjustment including femoral neck aBMD. In men, knee pain, rather than radiographic knee OA, was associated with multiple HR-pQCT structural and densitometric parameters (lower total and cortical vBMD, lower cortical thickness and larger trabecular area). Associations were largely absent at the distal radius, suggesting that weight-bearing sites may be more sensitive to knee OA-related bone alterations. Joint-specific analyses further suggested stronger patterns for patellofemoral OA compared with tibiofemoral OA.

### Potential Rationale for Radiographic Knee OA and Cortical vBMD in Women

We observed, in women only, an average 0.55 SD lower Ct. vBMD in those with radiographic knee OA, indicating a cortical-specific pattern. Sex-specific patterns in tibial cortical bone have also been described in prior knee OA studies, although the direction of associations has not been consistent across cohorts. A small study of 14 individuals (3 men, 11 women, age 50 ± 12 years) reported lower medial compartment vBMD at the proximal tibia of knee OA participants compared to non-OA controls [35]. A larger cohort study in the UK (295 men, age 65 ± 3 years; 288 women, age 67 ± 3 years) using pQCT found higher total and cortical area at the 14% distal tibia (a more proximal site than examined in the present study) was associated with higher KLG only in men, but not in women [21]. Cortical bone is a major contributor to long-bone stiffness and strength, and lower cortical density at a distal weight-bearing site may reflect broader structural differences in individuals with established knee OA. Our findings also contrast with the traditional view of OA as a condition associated with increased bone formation [36], suggesting that lower cortical bone density can coexist with radiographic disease. Subchondral bone changes are a well-described feature of knee OA [37–39], although we measured bone at a site remote from the knee, the association suggests that bone alterations in knee OA may extend beyond the subchondral region to a more distal location within the weight-bearing tibia.

### Potential Rationale for Knee Pain and HR-pQCT Parameters

We found that general knee pain assessed by BPI was associated with lower total vBMD, lower cortical vBMD, lower cortical thickness, and larger trabecular area at the distal tibia, most clearly in men and directionally similar (albeit with wider confidence intervals) in women. These findings are broadly consistent with prior evidence that symptomatic knee conditions may coincide with differences in bone quality at weight-bearing sites [8, 17, 22]. We suspect that bone microstructural deterioration associated with knee pain may be due to reduced physical activity. Although we adjusted for accelerometer-measured physical activity (mean steps per day), there is a possibility that residual differences in mechanical loading, including standing or other weight-bearing activities not captured may remain. Another explanation is that compromised cortical bone may co-occur with subchondral attrition, which is a recognized radiographic feature and risk factor for knee pain. The absence of corresponding associations at the distal radius suggests that weight-bearing sites may be more sensitive to pain-related differences in bone microarchitecture. Prior experimental and clinical studies have demonstrated that mechanical unloading can lead to rapid cortical bone loss at weight-bearing sites [40, 41]. Taken together, our findings suggest an association between altered bone microarchitectures at weight-bearing sites and activity-related knee pain. Our results also highlight that knee pain can be linked to bone parameters independently of femoral neck aBMD, suggesting that knee pain itself among knee OA patients may serve as a clinical warning for potential risk of compromised bone quality and subsequent fracture risk, beyond just radiographic disease severity alone.

### PF vs. TF Joint

We observed stronger evidence for compromised distal tibia bone microarchitecture for radiographic PF OA than for TF OA in women. The PF region is more engaged during stair climbing, deep flexion, and loading cycles that reach the knee and propagate to the tibia. Thus, structural involvement in PF OA might more readily influence distal skeletal loading and microarchitecture. In contrast, TF OA may represent localized cartilage or subchondral pathology without as strong a degree of mechanical coupling to distal bone to produce detectable microarchitectural signals. It is also possible that measurement error, misclassification, or lower statistical power in the TF-only stratum explains these weaker associations.

### Sex Differences

We observed sex-specific patterns whereby radiographic OA-related cortical deficits were more evident in women, while pain-related structural differences were primarily observed in men. These findings are consistent with known sex differences in bone biology and OA presentation. Women may be more likely to have cortical deficits under structural joint stress due to hormonal factors or lower baseline cortical bone reserve, whereas men may demonstrate stronger coupling of pain and disuse-driven biomechanical adaptation to systemic bone changes [42], although the mechanisms remain uncertain. Differences in hormonal environment, baseline cortical bone reserve, or behavioral responses to pain may contribute. Given limited power in sex-stratified analyses, these observations should be considered hypothesis-generating.

### Implications for Fracture Risk

An important rationale for this study was the paradox that individuals with knee OA often have higher aBMD yet experience increased fracture risk. Our findings help provide a potential microstructural explanation for this discrepancy. Several HR-pQCT measures identified in this study have been consistently associated with incident fragility fractures independent of aBMD and clinical risk factors in large prospective cohorts [15, 43, 44]. Thus, the cortical deficits observed in women with radiographic knee OA, and the broader structural deficits associated with knee pain in men, may represent skeletal features that are not captured by DXA but are relevant to fracture susceptibility.

These results support a conceptual model in which knee OA or pain may coexist with, or contribute to, the deterioration in cortical bone quality at weight-bearing sites, potentially increasing fragility despite preserved or elevated aBMD. Reduced loading due to pain-related activity modification, altered gait mechanics, or systemic remodeling processes could plausibly contribute to these changes. Although the cross-sectional design precludes causal inference, our findings suggest that bone microarchitecture may be an intermediate pathway linking OA symptoms to fracture risk. If confirmed longitudinally, HR-pQCT-derived cortical measures could refine fracture risk stratification among older adults with knee OA beyond standard DXA-based assessment.

### Strengths and Limitations

Our study has several strengths. We used HR-pQCT to assess bone microstructure and quality, providing separate details on trabecular and cortical bone and estimated bone strength that traditional DXA measures cannot offer. We were able to assess radiographic knee OA separately at the PF and TF joint, which allowed us to identify potentially distinct patterns of association between distal tibia bone microarchitecture and compartment-specific disease. Models were adjusted for major confounders including age, BMI, aBMD, physical activity, and medication use. We additionally examined multiple definitions of knee OA (radiographic, various pain measures and the combination) and performed sensitivity analyses, lending consistency to our findings.

Nevertheless, there are important limitations. First, the cross-sectional design precludes inference about temporality or causality, and it remains unclear whether altered bone microarchitecture precedes OA or results from OA-related changes in loading or activity. Second, our bone measurements were taken at the distal tibia and distal radius, not at the knee subchondral bone itself. While these peripheral sites are informative about overall bone health and fracture risk, they do not directly capture localized bone changes at the knee. Nevertheless, our results could play a hypothesis-generating role in understanding relationships between local and systemic bone structures in individuals with knee OA. Third, radiographic OA classification using KLG combines multiple structural features into a single score and thus may introduce heterogeneity when grouping KLG 3 and 4. Pain measures were self-reported and not specific to OA, and pain perception can be influenced by factors beyond joint structure. Fourth, sample sizes were modest within some sex-specific and sensitivity analyses, which may have limited statistical power. We evaluated multiple HR-pQCT outcomes and performed correction for multiple comparisons; however, because of the limited sample size and the high correlation across many bone parameters, strict reliance on adjusted p-values alone may overlook potentially meaningful associations. Therefore, findings should be interpreted based on overall patterns, effect sizes, and biological plausibility rather than statistical significance alone. Fifth, some potential confounders, such as prior knee trauma (e.g., cruciate ligament or meniscal injury) and lower-limb alignment, could not be adjusted for in the models due to data availability. Finally, participants were relatively healthy older adults, and this selection was further accentuated when restricting to older adults who participated in both ancillary studies. This may limit generalizability to more complex populations or those with very advanced knee OA who might already have undergone joint replacement. Despite these limitations, our study provides a detailed perspective on bone and joint relationships that generate new hypotheses relevant to future knee OA research.

## Conclusions

In conclusion, in this population of older adults, having established radiographic knee osteoarthritis was more strongly related to lower cortical density at the distal tibia in women, whereas the presence of knee pain was linked to deficits in overall structure and density in men. These sex-specific patterns may help explain variable fracture risk patterns in osteoarthritis populations and provide insight into sex-specific etiology. Longitudinal and experimental studies are needed to clarify the direction of causality and clinical implications of these microarchitectural measures on incident fragility fractures.

## Supplementary Information

Below is the link to the electronic supplementary material.

Supplementary Material 1

Supplementary Material 2

## References

[1] Lawrence RC et al (2008) Estimates of the prevalence of arthritis and other rheumatic conditions in the United States. Part II. Arthritis Rheum 58(1):26–35 18163497 10.1002/art.23176 PMC3266664

[2] GBD 2017 Disease and Injury Incidence and Prevalence Collaborators (2018) Global, regional, and national incidence, prevalence, and years lived with disability for 354 diseases and injuries for 195 countries and territories, 1990–2017: a systematic analysis for the global burden of disease study 2017. Lancet 392(10159):1789–1858 30496104 10.1016/S0140-6736(18)32279-7 PMC6227754

[3] Hunter DJ, Bierma-Zeinstra S (2019) Osteoarthritis. Lancet 393(10182):1745–1759 31034380 10.1016/S0140-6736(19)30417-9

[4] Arden NK et al (2006) Knee pain, knee osteoarthritis, and the risk of fracture. Arthritis Rheum 55(4):610–615 16874784 10.1002/art.22088

[5] Jacob L, Kostev K (2021) Osteoarthritis and the incidence of fracture in the United Kingdom: a retrospective cohort study of 258,696 patients. Osteoarthr Cartil 29(2):215–221 10.1016/j.joca.2020.12.006 33359250

[6] Bergink AP et al (2003) Osteoarthritis of the knee is associated with vertebral and nonvertebral fractures in the elderly: the Rotterdam Study. Arthritis Rheum 49(5):648–657 14558050 10.1002/art.11380

[7] Burr DB, Gallant MA (2012) Bone remodelling in osteoarthritis. Nat Rev Rheumatol 8(11):665–673 22868925 10.1038/nrrheum.2012.130

[8] Liew JW et al (2025) Relationship between knee pain and depth-specific measures of proximal tibial subchondral bone density. Osteoarthr Cartil 33(5):625–632 10.1016/j.joca.2025.02.781 PMC12034474 40089263

[9] Goldring SR, Goldring MB (2016) Changes in the osteochondral unit during osteoarthritis: structure, function and cartilage-bone crosstalk. Nat Rev Rheumatol 12(11):632–644 27652499 10.1038/nrrheum.2016.148

[10] Kim YH, Lee JS, Park JH (2018) Association between bone mineral density and knee osteoarthritis in Koreans: the Fourth and Fifth Korea National Health and Nutrition Examination Surveys. Osteoarthr Cartil 26(11):1511–1517 10.1016/j.joca.2018.07.008 30056213

[11] Barbour KE et al (2017) Bone mineral density and the risk of hip and knee osteoarthritis: the Johnston county osteoarthritis project. Arthritis Care Res (Hoboken) 69(12):1863–1870 28129489 10.1002/acr.23211 PMC5529272

[12] Fang L et al (2021) Defining disease progression in Chinese mainland people: association between bone mineral density and knee osteoarthritis. J Orthop Transl 26:39–44 10.1016/j.jot.2020.07.006 PMC7773972 33437621

[13] Anand V et al (2022) Study of relationship between bone mineral density in ipsilateral proximal femur and severity of osteoarthritis of knee. J Fam Med Prim Care 11(2):599–602 10.4103/jfmpc.jfmpc_1006_21 PMC8963643 35360774

[14] Choi ES et al (2021) Relationship of bone mineral density and knee osteoarthritis (Kellgren–Lawrence grade): fifth Korea National Health and Nutrition Examination Survey. Clin Orthop Surg 13(1):60–66 33747379 10.4055/cios20111 PMC7948046

[15] Samelson EJ et al (2019) Cortical and trabecular bone microarchitecture as an independent predictor of incident fracture risk in older women and men in the Bone Microarchitecture International Consortium (BoMIC): a prospective study. Lancet Diabetes Endocrinol 7(1):34–43 30503163 10.1016/S2213-8587(18)30308-5 PMC6354581

[16] Kroker A et al (2017) Quantitative in vivo assessment of bone microarchitecture in the human knee using HR-pQCT. Bone 97:43–48 28039095 10.1016/j.bone.2016.12.015

[17] Bennell KL et al (2008) Tibial subchondral trabecular volumetric bone density in medial knee joint osteoarthritis using peripheral quantitative computed tomography technology. Arthritis Rheum 58(9):2776–2785 18759296 10.1002/art.23795

[18] Besler BA et al (2021) Bone and joint enhancement filtering: application to proximal femur segmentation from uncalibrated computed tomography datasets. Med Image Anal 67:101887 33181434 10.1016/j.media.2020.101887

[19] Boyd SK (2024) High-resolution peripheral quantitative computed tomography in rheumatic diseases: a new option for knee osteoarthritis. Radiol Clin N Am 62(5):903–912 39059980 10.1016/j.rcl.2024.02.010

[20] Wong A, Costa S, Anwari V, Liu S, Hernandez ME, Cagnoni A, Naraghi A, Mohankumar R, Sussman M, Johnston JD, Giangregorio L (2024) Higher subchondral fluid fraction but lower marrow density are related to weaker bones and stronger knee symptoms in postmenopausal knee osteoarthritis [Conference presentation abstract]. J Bone Miner Res 39(Suppl 1):S239

[21] Abdin-Mohamed M et al (2009) Volumetric bone mineral density of the tibia is not increased in subjects with radiographic knee osteoarthritis. Osteoarthr Cartil 17(2):174–177 10.1016/j.joca.2008.06.004 18684648

[22] Burnett WD et al (2017) Proximal tibial trabecular bone mineral density is related to pain in patients with osteoarthritis. Arthritis Res Ther 19(1):200 28899428 10.1186/s13075-017-1415-9 PMC5596910

[23] Cummings SR et al (2023) The study of muscle, mobility and aging (SOMMA). A unique cohort study about the cellular biology of aging and age-related loss of mobility. J Gerontol A Biol Sci Med Sci 78:2083–2093 36754371 10.1093/gerona/glad052 PMC10613002

[24] SOMMA study website. Available from: https://www.sommastudy.com/

[25] Mau T et al (2022) Mitochondrial energetics in skeletal muscle are associated with leg power and cardiorespiratory fitness in the study of muscle, mobility, and aging (SOMMA). J Gerontol A Biol Sci Med Sci 78:1367–1375 10.1093/gerona/glac238 PMC10395564 36462195

[26] Bonaretti S et al (2017) The comparability of HR-pQCT bone measurements is improved by scanning anatomically standardized regions. Osteoporos Int 28(7):2115–2128 28391447 10.1007/s00198-017-4010-7 PMC5526099

[27] Whittier DE et al (2020) Guidelines for the assessment of bone density and microarchitecture in vivo using high-resolution peripheral quantitative computed tomography. Osteoporos Int 31(9):1607–1627 32458029 10.1007/s00198-020-05438-5 PMC7429313

[28] Pauchard Y et al (2012) Quality control for bone quality parameters affected by subject motion in high-resolution peripheral quantitative computed tomography. Bone 50(6):1304–1310 22445540 10.1016/j.bone.2012.03.003

[29] Manske SL et al (2015) Human trabecular bone microarchitecture can be assessed independently of density with second generation HR-pQCT. Bone 79:213–221 26079995 10.1016/j.bone.2015.06.006

[30] Cheung AM et al (2013) High-resolution peripheral quantitative computed tomography for the assessment of bone strength and structure: a review by the Canadian Bone Strength Working Group. Curr Osteoporos Rep 11(2):136–146 23525967 10.1007/s11914-013-0140-9 PMC3641288

[31] Pistoia W et al (2002) Estimation of distal radius failure load with micro-finite element analysis models based on three-dimensional peripheral quantitative computed tomography images. Bone 30(6):842–848 12052451 10.1016/s8756-3282(02)00736-6

[32] Kothari M et al (2004) Fixed-flexion radiography of the knee provides reproducible joint space width measurements in osteoarthritis. Eur Radiol 14(9):1568–1573 15150666 10.1007/s00330-004-2312-6

[33] Kellgren JH, Lawrence JS (1957) Radiological assessment of osteo-arthrosis. Ann Rheum Dis 16(4):494–502 13498604 10.1136/ard.16.4.494 PMC1006995

[34] Distefano G et al (2024) Skeletal muscle composition, power, and mitochondrial energetics in older men and women with knee osteoarthritis. Arthritis Rheumatol 76(12):1764–1774 39016102 10.1002/art.42953 PMC11605275

[35] Arjmand H et al (2018) Mechanical metrics of the proximal tibia are precise and differentiate osteoarthritic and normal knees: a finite element study. Sci Rep 8(1):11478 30065276 10.1038/s41598-018-29880-y PMC6068127

[36] Im GI, Kim MK (2014) The relationship between osteoarthritis and osteoporosis. J Bone Miner Metab 32(2):101–109 24196872 10.1007/s00774-013-0531-0

[37] Castañeda S et al (2012) Subchondral bone as a key target for osteoarthritis treatment. Biochem Pharmacol 83(3):315–323 21964345 10.1016/j.bcp.2011.09.018

[38] Radin EL, Rose RM (1986) Role of subchondral bone in the initiation and progression of cartilage damage. Clin Orthop Relat Res 213:34–40 3780104

[39] Burr DB (2004) The importance of subchondral bone in the progression of osteoarthritis. J Rheumatol Suppl 70:77–80 15132360

[40] Kazakia GJ et al (2014) The influence of disuse on bone microstructure and mechanics assessed by HR-pQCT. Bone 63:132–140 24603002 10.1016/j.bone.2014.02.014 PMC4041600

[41] Rittweger J et al (2009) Bone loss in the lower leg during 35 days of bed rest is predominantly from the cortical compartment. Bone 44(4):612–618 19168165 10.1016/j.bone.2009.01.001

[42] Zhang YY et al (2024) Insights and implications of sexual dimorphism in osteoporosis. Bone Res 12(1):8 38368422 10.1038/s41413-023-00306-4 PMC10874461

[43] Szulc P et al (2024) Fracture risk based on high-resolution peripheral quantitative computed tomography measures does not vary with age in older adults-the bone microarchitecture international consortium prospective cohort study. J Bone Miner Res 39(5):561–570 38477737 10.1093/jbmr/zjae033 PMC11205894

[44] Langsetmo L et al (2018) Volumetric bone mineral density and failure load of distal limbs predict incident clinical fracture independent HR-pQCT BMD and failure load predicts incident clinical fracture of FRAX and clinical risk factors among older men. J Bone Miner Res 33(7):1302–1311 29624722 10.1002/jbmr.3433 PMC6048962

