# Supplementary material for: Sex-Specific Associations of Radiographic Knee Osteoarthritis and Pain with Distal Tibia Bone Microarchitecture: the Study of Muscle, Mobility and Aging (SOMMA)
Source: Calcif Tissue Int. 2026 Apr 27;117(1):65. doi: 10.1007/s00223-026-01531-9 (PMC13111493; doi:10.1007/s00223-026-01531-9)
Supplement: Supplementary file 1 — Supplementary Material 1 [file 223_2026_1531_MOESM1_ESM.docx]

**Supplementary Table 1. Participant characteristics in the SOMMA Bone and Knee OA bone ancillary studies by sex and general knee pain by BPI.**

|  | **Men** | | | | **Women** | | | |
| --- | --- | --- | --- | --- | --- | --- | --- | --- |
|  | **No BPI**  **knee pain** | **BPI knee pain** | **P-value** | **N** | **No BPI knee pain** | **BPI knee pain** | **P-value** | **N** |
|  | ***N=91*** | ***N=17*** |  |  | ***N=123*** | ***N=32*** |  |  |
| Age (years) | 76.0 [74.0;79.5] | 77.0 [74.0;81.0] | 0.573 | 108 | 76.0 [74.0;80.0] | 77.0 [74.0;82.2] | 0.436 | 155 |
| White race | 84 (92.3%) | 16 (94.1%) | 1.000 | 108 | 102 (82.9%) | 26 (81.2%) | 1.000 | 155 |
| **BMI (kg/m^2^)** | **27.2 (4.1)** | **29.6 (4.1)** | **0.041** | **108** | **27.2 (4.8)** | **29.8 (5.0)** | **0.012** | **155** |
| **Weight, kg** | **81.1 (12.8)** | **91.8 (14.4)** | **0.010** | **108** | **69.0 (12.7)** | **76.5 (11.5)** | **0.002** | **155** |
| Height, m | 1.7 (0.1) | 1.8 (0.1) | 0.054 | 108 | 1.6 (0.1) | 1.6 (0.1) | 0.243 | 155 |
| Income level: |  |  | 0.770 | 102 |  |  | 0.423 | 138 |
| < $50,000 | 24 (27.9%) | 5 (31.2%) |  |  | 55 (50.0%) | 17 (60.7%) |  |  |
| ≥ $50,000 | 62 (72.1%) | 11 (68.8%) |  |  | 55 (50.0%) | 11 (39.3%) |  |  |
| Fracture since age 50 | 10 (11.0%) | 4 (23.5%) | 0.229 | 108 | **33 (26.8%)** | **15 (48.4%)** | **0.036** | **154** |
| Ever smoker | 43 (47.3%) | 8 (47.1%) | 1.000 | 108 | 56 (45.5%) | 13 (41.9%) | 0.875 | 154 |
| Alcohol drink per week: |  |  | 0.789 | 108 |  |  | 0.893 | 151 |
| No drinks | 28 (30.8%) | 6 (35.3%) |  |  | 63 (52.5%) | 17 (54.8%) |  |  |
| 0-2 drink | 29 (31.9%) | 4 (23.5%) |  |  | 28 (23.3%) | 6 (19.4%) |  |  |
| ≥3 drinks | 34 (37.4%) | 7 (41.2%) |  |  | 29 (24.2%) | 8 (25.8%) |  |  |
| Physical activity (steps per day) | **7059.3 (3489.6)** | **5439.1 (2237.1)** | **0.040** | **83** | **7051.0 (3299.8)** | **5179.6 (2306.4)** | **0.002** | **125** |
| Multi-comorbidity index | 1.0 [0.0;1.0] | 0.0 [0.0;1.0] | 0.094 | 107 | 0.0 [0.0;1.0] | 1.0 [0.0;1.0] | 0.157 | 153 |
| Osteoporosis medication | 1 (1.1%) | 0 (0.0%) | 1.000 | 108 | 17 (13.8%) | 3 (9.4%) | 0.767 | 155 |
| Prescription pain medication | 7 (7.7%) | 0 (0.0%) | 0.594 | 108 | 15 (12.2%) | 5 (15.6%) | 0.565 | 155 |
| Femoral neck BMD (g/cm^2^) | 0.9 (0.1) | 1.0 (0.2) | 0.675 | 108 | 0.8 (0.1) | 0.8 (0.1) | 0.521 | 155 |
|  |  |  |  |  |  |  |  |  |

^a^ Abbreviations: BPI = Brief Pain Inventory, N = number, CI = confidence interval

^b^ Bold text: statistically significant (p<0.05)

**Supplementary Table 2a Associations between standardized HR-pQCT parameters at the distal radius (DR) and radiographic knee OA (as determined by KLG, with levels 3-4 indicative of advanced disease) in the SOMMA Bone and Knee OA ancillary studies as determined from multivariable linear regression models.**

|  | Radiographic knee OA: KLG 3-4 vs. 0-2 | | | | | |
| --- | --- | --- | --- | --- | --- | --- |
|  | Men (N=75) | | | Women (N=108) | | |
| **DR HR-pQCT** | B | 95% CI | p-value | B | 95% CI | p-value |
| Tt. vBMD | 0.02 | (-0.46,0.51) | 0.922 | -0.12 | (-0.53,0.30) | 0.576 |
| Tt. Area | -0.23 | (-0.85,0.38) | 0.462 | -0.10 | (-0.57,0.36) | 0.666 |
| Ct. vBMD | -0.09 | (-0.67,0.50) | 0.770 | -0.42 | (-0.86,0.02) | 0.066 |
| Ct. Area | 0.05 | (-0.49,0.59) | 0.855 | -0.40 | (-0.80,0.01) | 0.058 |
| Ct. Th | -0.01 | (-0.54,0.53) | 0.977 | -0.30 | (-0.74,0.14) | 0.185 |
| Tb. vBMD | 0.04 | (-0.40,0.47) | 0.871 | 0.12 | (-0.27,0.51) | 0.561 |
| Tb. Area | -0.18 | (-0.73,0.38) | 0.530 | 0.01 | (-0.45,0.47) | 0.967 |
| Tb. Th | -0.19 | (-0.68,0.30) | 0.457 | 0.28 | (-0.14,0.69) | 0.193 |
| Tb. N | 0.33 | (-0.15,0.80) | 0.183 | 0.01 | (-0.37,0.39) | 0.940 |
| FEA failure load | -0.06 | (-0.54,0.42) | 0.816 | -0.23 | (-0.65,0.18) | 0.278 |

^a^ Abbreviations: OA = osteoarthritis, KLG = Kellgren Lawrence Grade, Tt. = total, Ct. = cortical, Tb. = trabecular, FEA = finite element analysis, Th = thickness, N = number, CI = confidence interval

^b^ Model adjustments: age, BMI, fracture since age 50, smoking (ever vs. never), drinking (≥2, 0-2, vs. 0 drinks/week), physical activity (ActivPAL4^TM^, steps/day), femoral neck BMD T-score, osteoporosis medication.

**Supplementary Table 2b Associations between standardized HR-pQCT parameters at the distal tibia (DT) and radiographic knee OA (as determined by KLG, with levels 2-4 indicative of any disease) in the SOMMA Bone and Knee OA ancillary studies as determined from multivariable linear regression models.**

|  | Radiographic knee OA: KLG 2-4 vs. 0-1 | | | | | |
| --- | --- | --- | --- | --- | --- | --- |
|  | Men (N=82) | | | Women (N=121) | | |
| **DT HR-pQCT** | B | 95% CI | p-value | B | 95% CI | p-value |
| Tt. vBMD | -0.39 | (-0.87,0.09) | 0.119 | 0.03 | (-0.33,0.39) | 0.870 |
| Tt. Area | 0.47 | (-0.05,1.00) | 0.082 | -0.15 | (-0.55,0.25) | 0.454 |
| Ct. vBMD | -0.14 | (-0.65,0.36) | 0.587 | -0.13 | (-0.50,0.24) | 0.508 |
| Ct. Area | -0.29 | (-0.73,0.16) | 0.213 | 0.08 | (-0.27,0.43) | 0.647 |
| Ct. Th | -0.42 | (-0.90,0.06) | 0.089 | 0.09 | (-0.28,0.47) | 0.625 |
| Tb. vBMD | -0.10 | (-0.54,0.34) | 0.652 | -0.06 | (-0.44,0.32) | 0.745 |
| Tb. Area | 0.43 | (-0.08,0.95) | 0.105 | -0.09 | (-0.50,0.31) | 0.660 |
| Tb. Th | -0.15 | (-0.63,0.33) | 0.533 | -0.10 | (-0.52,0.32) | 0.629 |
| Tb. N | -0.16 | (-0.67,0.35) | 0.535 | 0.07 | (-0.34,0.47) | 0.753 |
| FEA failure load | -0.04 | (-0.41,0.34) | 0.841 | -0.06 | (-0.37,0.26) | 0.713 |

^a^ Abbreviations: OA = osteoarthritis, KLG = Kellgren Lawrence Grade, Tt. = total, Ct. = cortical, Tb. = trabecular, FEA = finite element analysis, Th = thickness, N = number, CI = confidence interval

^b^ Model adjustments: age, BMI, fracture since age 50, smoking (ever vs. never), drinking (≥2, 0-2, vs. 0 drinks/week), physical activity (ActivPAL4^TM^, steps/day), femoral neck BMD T-score, osteoporosis medication.

**Supplementary Table 2c Associations between standardized HR-pQCT parameters at the distal radius (DR) and radiographic knee OA (as determined by KLG, with levels 2-4 indicative of any disease) in the SOMMA Bone and Knee OA ancillary studies as determined from multivariable linear regression models.**

|  | Radiographic knee OA: KLG 2-4 vs. 0-1 | | | | | |
| --- | --- | --- | --- | --- | --- | --- |
|  | Men (N=75) | | | Women (N=108) | | |
| **DR HR-pQCT** | B | 95% CI | p-value | B | 95% CI | p-value |
| Tt. vBMD | -0.29 | (-0.73,0.16) | 0.212 | -0.25 | (-0.66,0.16) | 0.242 |
| Tt. Area | 0.30 | (-0.27,0.88) | 0.308 | -0.11 | (-0.57,0.36) | 0.651 |
| Ct. vBMD | -0.24 | (-0.79,0.30) | 0.382 | -0.01 | (-0.45,0.44) | 0.978 |
| Ct. Area | -0.17 | (-0.67,0.33) | 0.507 | -0.14 | (-0.55,0.26) | 0.493 |
| Ct. Th | -0.33 | (-0.82,0.17) | 0.198 | -0.11 | (-0.55,0.33) | 0.626 |
| Tb. vBMD | -0.22 | (-0.62,0.18) | 0.281 | -0.30 | (-0.68,0.09) | 0.130 |
| Tb. Area | 0.22 | (-0.30,0.74) | 0.405 | -0.06 | (-0.52,0.39) | 0.786 |
| Tb. Th | -0.22 | (-0.68,0.24) | 0.348 | -0.26 | (-0.68,0.15) | 0.221 |
| Tb. N | 0.01 | (-0.44,0.46) | 0.974 | -0.23 | (-0.61,0.15) | 0.232 |
| FEA failure load | -0.31 | (-0.75,0.14) | 0.179 | -0.23 | (-0.64,0.19) | 0.284 |

^a^ Abbreviations: OA = osteoarthritis, KLG = Kellgren Lawrence Grade, Tt. = total, Ct. = cortical, Tb. = trabecular, FEA = finite element analysis, Th = thickness, N = number, CI = confidence interval

^b^ Model adjustments: age, BMI, fracture since age 50, smoking (ever vs. never), drinking (≥2, 0-2, vs. 0 drinks/week), physical activity (ActivPAL4^TM^, steps/day), femoral neck BMD T-score, osteoporosis medication.

**Supplementary Table 3a Associations between standardized HR-pQCT parameters at the distal radius (DR) and general knee pain by BPI in the SOMMA Bone and Knee OA ancillary studies as determined from multivariable linear regression models.**

|  | BPI knee pain (yes vs. no) | | | | | |
| --- | --- | --- | --- | --- | --- | --- |
|  | Men (N=76) | | | Women (N=111) | | |
| **DR HR-pQCT** | B | 95% CI | p-value | B | 95% CI | p-value |
| Tt. vBMD | -0.12 | (-0.73,0.48) | 0.687 | 0.06 | (-0.40,0.52) | 0.784 |
| Tt. Area | -0.15 | (-0.90,0.61) | 0.707 | -0.38 | (-0.89,0.13) | 0.149 |
| Ct. vBMD | -0.11 | (-0.82,0.60) | 0.764 | 0.32 | (-0.18,0.81) | 0.214 |
| Ct. Area | 0.49 | (-0.14,1.13) | 0.133 | -0.20 | (-0.66,0.25) | 0.385 |
| Ct. Th | 0.19 | (-0.47,0.85) | 0.573 | -0.00 | (-0.49,0.49) | 0.996 |
| Tb. vBMD | -0.21 | (-0.71,0.30) | 0.425 | -0.00 | (-0.44,0.43) | 0.986 |
| Tb. Area | 0.25 | (-0.44,0.95) | 0.475 | -0.25 | (-0.75,0.25) | 0.323 |
| Tb. Th | -0.03 | (-0.62,0.56) | 0.916 | -0.15 | (-0.62,0.31) | 0.522 |
| Tb. N | -0.25 | (-0.82,0.32) | 0.390 | -0.04 | (-0.47,0.38) | 0.846 |
| FEA failure load | 0.34 | (-0.22,0.91) | 0.241 | -0.04 | (-0.50,0.43) | 0.872 |

^a^ Abbreviations: OA = osteoarthritis, BPI = Brief Pain Inventory, Tt. = total, Ct. = cortical, Tb. = trabecular, FEA = finite element analysis, Th = thickness, N = number, CI = confidence interval

^b^ Model adjustments: age, BMI, fracture since age 50, smoking (ever vs. never), drinking (≥2, 0-2, vs. 0 drinks/week), physical activity (ActivPAL4^TM^, steps/day), femoral neck BMD T-score, pain medication.

**Supplementary Table 3b Associations between standardized HR-pQCT parameters at the distal tibia (DT) and persistent general knee pain by BPI in the SOMMA Bone and Knee OA ancillary studies as determined from multivariable linear regression models.**

|  | BPI knee pain (yes vs. no) | | | | | |
| --- | --- | --- | --- | --- | --- | --- |
|  | Men (N=83) | | | Women (N=122) | | |
| **DT HR-pQCT** | B | 95% CI | p-value | B | 95% CI | p-value |
| Tt. vBMD | -0.14 | (-1.18,0.90) | 0.796 | -0.52 | (-1.35,0.32) | 0.230 |
| Tt. Area | 0.02 | (-1.10,1.14) | 0.973 | 0.08 | (-0.86,1.02) | 0.868 |
| Ct. vBMD | 0.03 | (-1.06,1.12) | 0.956 | -0.75 | (-1.61,0.12) | 0.096 |
| Ct. Area | 0.04 | (-0.93,1.01) | 0.930 | -0.49 | (-1.31,0.32) | 0.239 |
| Ct. Th | -0.14 | (-1.18,0.91) | 0.800 | -0.47 | (-1.34,0.41) | 0.296 |
| Tb. vBMD | -0.02 | (-0.96,0.92) | 0.972 | -0.14 | (-1.03,0.76) | 0.765 |
| Tb. Area | 0.07 | (-1.05,1.18) | 0.903 | 0.18 | (-0.77,1.13) | 0.713 |
| Tb. Th | 0.59 | (-0.43,1.61) | 0.262 | -0.04 | (-1.03,0.95) | 0.938 |
| Tb. N | 0.05 | (-1.05,1.15) | 0.932 | 0.10 | (-0.86,1.07) | 0.834 |
| FEA failure load | 0.08 | (-0.72,0.88) | 0.843 | -0.51 | (-1.24,0.22) | 0.176 |

^a^ Abbreviations: OA = osteoarthritis, BPI = Brief Pain Inventory, Tt. = total, Ct. = cortical, Tb. = trabecular, FEA = finite element analysis, Th = thickness, N = number, CI = confidence interval

^b^ Model adjustments: age, BMI, fracture since age 50, smoking (ever vs. never), drinking (≥2, 0-2, vs. 0 drinks/week), physical activity (ActivPAL4^TM^, steps/day), femoral neck BMD T-score, pain medication.

**Supplementary Table 3c Associations between standardized HR-pQCT parameters at the distal tibia (DR) and persistent general knee pain by BPI in the SOMMA Bone and Knee OA ancillary studies as determined from multivariable linear regression models.**

|  | BPI knee pain (yes vs. no) | | | | | |
| --- | --- | --- | --- | --- | --- | --- |
|  | Men (N=76) | | | Women (N=111) | | |
| **DR HR-pQCT** | B | 95% CI | p-value | B | 95% CI | p-value |
| Tt. vBMD | 0.73 | (-0.24,1.70) | 0.144 | 0.23 | (-0.62,1.08) | 0.597 |
| Tt. Area | -0.67 | (-1.89,0.55) | 0.285 | -0.58 | (-1.52,0.37) | 0.233 |
| Ct. vBMD | 0.37 | (-0.79,1.53) | 0.537 | 0.28 | (-0.64,1.21) | 0.545 |
| Ct. Area | **1.58** | **(0.60,2.57)** | **0.002*** | -0.20 | (-1.05,0.65) | 0.646 |
| Ct. Th | **1.09** | **(0.04,2.13)** | **0.045** | 0.24 | (-0.66,1.15) | 0.598 |
| Tb. vBMD | 0.42 | (-0.41,1.24) | 0.326 | -0.11 | (-0.91,0.69) | 0.787 |
| Tb. Area | -0.44 | (-1.57,0.69) | 0.451 | -0.75 | (-1.67,0.16) | 0.111 |
| Tb. Th | 0.90 | (-0.04,1.84) | 0.066 | -0.22 | (-1.08,0.65) | 0.623 |
| Tb. N | 0.08 | (-0.85,1.01) | 0.862 | 0.14 | (-0.65,0.92) | 0.735 |
| FEA failure load | **1.20** | **(0.31,2.10)** | **0.010*** | -0.41 | (-1.27,0.44) | 0.345 |

^a^ Abbreviations: OA = osteoarthritis, BPI = Brief Pain Inventory, Tt. = total, Ct. = cortical, Tb. = trabecular, FEA = finite element analysis, Th = thickness, N = number, CI = confidence interval

^b^ Model adjustments: age, BMI, fracture since age 50, smoking (ever vs. never), drinking (≥2, 0-2, vs. 0 drinks/week), physical activity (ActivPAL4^TM^, steps/day), femoral neck BMD T-score, pain medication.

^c^ Bold text: statistically significant (p<0.05)

^d^ * denotes p < 0.05 after accounting for multiple comparison.

**Supplementary Table 4a Associations between standardized HR-pQCT parameters at the distal radius (DR) and functional knee pain when climbing stairs in the SOMMA Bone and Knee OA ancillary studies as determined from multivariable linear regression models.**

|  | Knee pain when climbing stairs (yes vs. no) | | | | | |
| --- | --- | --- | --- | --- | --- | --- |
|  | Men (N=83) | | | Women (N=122) | | |
| **DR HR-pQCT** | B | 95% CI | p-value | B | 95% CI | p-value |
| Tt. vBMD | 0.10 | (-0.40,0.60) | 0.704 | 0.07 | (-0.31,0.44) | 0.729 |
| Tt. Area | -0.33 | (-0.87,0.21) | 0.231 | -0.09 | (-0.51,0.33) | 0.682 |
| Ct. vBMD | -0.11 | (-0.63,0.42) | 0.694 | 0.32 | (-0.07,0.71) | 0.110 |
| Ct. Area | **-0.49** | **(-0.94,-0.03)** | **0.039** | 0.18 | (-0.18,0.55) | 0.329 |
| Ct. Th | -0.22 | (-0.73,0.28) | 0.394 | 0.16 | (-0.23,0.55) | 0.433 |
| Tb. vBMD | 0.35 | (-0.10,0.79) | 0.133 | -0.16 | (-0.56,0.24) | 0.437 |
| Tb. Area | -0.36 | (-0.89,0.18) | 0.196 | -0.15 | (-0.58,0.27) | 0.487 |
| Tb. Th | **0.55** | **(0.07,1.04)** | **0.028** | -0.19 | (-0.63,0.25) | 0.402 |
| Tb. N | 0.31 | (-0.22,0.84) | 0.252 | 0.14 | (-0.29,0.58) | 0.518 |
| FEA failure load | -0.31 | (-0.69,0.07) | 0.117 | -0.06 | (-0.39,0.27) | 0.715 |

^a^ Abbreviations: OA = osteoarthritis, Tt. = total, Ct. = cortical, Tb. = trabecular, FEA = finite element analysis, Th = thickness, N = number, CI = confidence interval

^b^ Model adjustments: age, BMI, fracture since age 50, smoking (ever vs. never), drinking (≥2, 0-2, vs. 0 drinks/week), physical activity (ActivPAL4^TM^, steps/day), femoral neck BMD T-score, pain medication.

^c^ Bold text: statistically significant (p<0.05)

**Supplementary Table 4b Associations between standardized HR-pQCT parameters at the distal tibia (DT) and persistent functional knee pain when climbing stairs in the SOMMA Bone and Knee OA ancillary studies as determined from multivariable linear regression models.**

|  | Knee pain when climbing stairs (yes vs. no) | | | | | |
| --- | --- | --- | --- | --- | --- | --- |
|  | Men (N=83) | | | Women (N=122) | | |
| **DT HR-pQCT** | B | 95% CI | p-value | B | 95% CI | p-value |
| Tt. vBMD | -0.46 | (-1.09,0.17) | 0.157 | -0.01 | (-0.44,0.41) | 0.946 |
| Tt. Area | -0.09 | (-0.79,0.60) | 0.795 | -0.18 | (-0.66,0.29) | 0.453 |
| Ct. vBMD | -0.23 | (-0.90,0.43) | 0.493 | 0.30 | (-0.14,0.75) | 0.187 |
| Ct. Area | **-0.61** | **(-1.19,-0.03)** | **0.044** | 0.16 | (-0.25,0.58) | 0.444 |
| Ct. Th | -0.42 | (-1.06,0.22) | 0.205 | 0.20 | (-0.24,0.65) | 0.374 |
| Tb. vBMD | -0.31 | (-0.89,0.26) | 0.288 | -0.29 | (-0.74,0.16) | 0.213 |
| Tb. Area | -0.03 | (-0.72,0.66) | 0.937 | -0.19 | (-0.68,0.29) | 0.438 |
| Tb. Th | 0.13 | (-0.51,0.76) | 0.696 | -0.14 | (-0.64,0.36) | 0.590 |
| Tb. N | -0.29 | (-0.97,0.38) | 0.401 | -0.17 | (-0.66,0.32) | 0.495 |
| FEA failure load | **-0.56** | **(-1.04,-0.09)** | **0.023** | -0.14 | (-0.52,0.23) | 0.453 |

^a^ Abbreviations: OA = osteoarthritis, Tt. = total, Ct. = cortical, Tb. = trabecular, FEA = finite element analysis, Th = thickness, N = number, CI = confidence interval

^b^ Model adjustments: age, BMI, fracture since age 50, smoking (ever vs. never), drinking (≥2, 0-2, vs. 0 drinks/week), physical activity (ActivPAL4^TM^, steps/day), femoral neck BMD T-score, pain medication.

^c^ Bold text: statistically significant (p<0.05)

**Supplementary Table 4c Associations between standardized HR-pQCT parameters at the distal radius (DR) and persistent functional knee pain when climbing stairs in the SOMMA Bone and Knee OA ancillary studies as determined from multivariable linear regression models.**

|  | Knee pain when climbing stairs (yes vs. no) | | | | | |
| --- | --- | --- | --- | --- | --- | --- |
|  | Men (N=76) | | | Women (N=111) | | |
| **DR HR-pQCT** | B | 95% CI | p-value | B | 95% CI | p-value |
| Tt. vBMD | -0.13 | (-0.84,0.57) | 0.711 | -0.25 | (-0.74,0.24) | 0.326 |
| Tt. Area | 0.17 | (-0.71,1.05) | 0.709 | 0.10 | (-0.46,0.65) | 0.735 |
| Ct. vBMD | -0.43 | (-1.25,0.39) | 0.310 | 0.11 | (-0.42,0.65) | 0.677 |
| Ct. Area | 0.06 | (-0.69,0.81) | 0.874 | -0.16 | (-0.65,0.34) | 0.537 |
| Ct. Th | -0.25 | (-1.01,0.52) | 0.532 | -0.21 | (-0.74,0.31) | 0.433 |
| Tb. vBMD | -0.02 | (-0.61,0.58) | 0.957 | -0.40 | (-0.86,0.06) | 0.091 |
| Tb. Area | 0.31 | (-0.50,1.11) | 0.455 | -0.01 | (-0.55,0.53) | 0.958 |
| Tb. Th | 0.18 | (-0.51,0.86) | 0.619 | -0.30 | (-0.80,0.20) | 0.237 |
| Tb. N | 0.04 | (-0.62,0.71) | 0.900 | -0.24 | (-0.69,0.21) | 0.303 |
| FEA failure load | -0.05 | (-0.72,0.62) | 0.891 | -0.33 | (-0.82,0.17) | 0.198 |

^a^ Abbreviations: OA = osteoarthritis, Tt. = total, Ct. = cortical, Tb. = trabecular, FEA = finite element analysis, Th = thickness, N = number, CI = confidence interval

^b^ Model adjustments: age, BMI, fracture since age 50, smoking (ever vs. never), drinking (≥2, 0-2, vs. 0 drinks/week), physical activity (ActivPAL4^TM^, steps/day), femoral neck BMD T-score, pain medication.

**Supplementary Table 5a Associations between standardized HR-pQCT parameters at the distal radius (DR) and functional knee pain when walking along a flat surface in the SOMMA Bone and Knee OA ancillary studies as determined from multivariable linear regression models.**

|  | knee pain when walking flat (yes vs. no) | | | | | |
| --- | --- | --- | --- | --- | --- | --- |
|  | Men (N=76) | | | Women (N=111) | | |
| **DR HR-pQCT** | B | 95% CI | p-value | B | 95% CI | p-value |
| Tt. vBMD | -0.23 | (-0.92,0.45) | 0.509 | -0.22 | (-0.75,0.31) | 0.419 |
| Tt. Area | -0.26 | (-1.12,0.59) | 0.548 | 0.17 | (-0.42,0.77) | 0.573 |
| Ct. vBMD | -0.21 | (-1.02,0.60) | 0.612 | 0.19 | (-0.39,0.77) | 0.530 |
| Ct. Area | -0.14 | (-0.87,0.59) | 0.709 | -0.08 | (-0.61,0.46) | 0.783 |
| Ct. Th | -0.22 | (-0.97,0.53) | 0.561 | -0.04 | (-0.61,0.53) | 0.897 |
| Tb. vBMD | -0.22 | (-0.79,0.36) | 0.466 | -0.24 | (-0.74,0.26) | 0.356 |
| Tb. Area | -0.06 | (-0.85,0.73) | 0.883 | 0.06 | (-0.53,0.64) | 0.849 |
| Tb. Th | -0.11 | (-0.79,0.56) | 0.745 | 0.01 | (-0.53,0.56) | 0.958 |
| Tb. N | -0.33 | (-0.97,0.31) | 0.319 | -0.21 | (-0.70,0.28) | 0.401 |
| FEA failure load | 0.00 | (-0.65,0.66) | 0.992 | -0.16 | (-0.70,0.37) | 0.551 |

^a^ Abbreviations: OA = osteoarthritis, Tt. = total, Ct. = cortical, Tb. = trabecular, FEA = finite element analysis, Th = thickness, N = number, CI = confidence interval

^b^ Model adjustments: age, BMI, fracture since age 50, smoking (ever vs. never), drinking (≥2, 0-2, vs. 0 drinks/week), physical activity (ActivPAL4^TM^, steps/day), femoral neck BMD T-score, pain medication.

**Supplementary Table 5b Associations between standardized HR-pQCT parameters at the distal tibia (DT) and persistent functional knee pain when walking along a flat surface in the SOMMA Bone and Knee OA ancillary studies as determined from multivariable linear regression models.**

|  | knee pain when walking flat (yes vs. no) | | | | | |
| --- | --- | --- | --- | --- | --- | --- |
|  | Men (N=76) | | | Women (N=111) | | |
| **DT HR-pQCT** | B | 95% CI | p-value | B | 95% CI | p-value |
| Tt. vBMD | -0.10 | (-1.25,1.05) | 0.861 | 0.32 | (-0.28,0.93) | 0.296 |
| Tt. Area | -0.52 | (-1.76,0.72) | 0.410 | -0.49 | (-1.16,0.18) | 0.154 |
| Ct. vBMD | -0.35 | (-1.55,0.85) | 0.570 | 0.28 | (-0.35,0.92) | 0.381 |
| Ct. Area | 0.07 | (-1.00,1.15) | 0.895 | 0.46 | (-0.13,1.04) | 0.128 |
| Ct. Th | 0.23 | (-0.93,1.40) | 0.693 | 0.56 | (-0.07,1.18) | 0.084 |
| Tb. vBMD | -0.15 | (-1.19,0.89) | 0.776 | -0.15 | (-0.80,0.50) | 0.649 |
| Tb. Area | -0.47 | (-1.70,0.76) | 0.460 | -0.46 | (-1.14,0.22) | 0.191 |
| Tb. Th | 0.84 | (-0.29,1.96) | 0.151 | 0.39 | (-0.32,1.10) | 0.284 |
| Tb. N | -1.03 | (-2.22,0.17) | 0.096 | -0.08 | (-0.78,0.62) | 0.823 |
| FEA failure load | -0.21 | (-1.09,0.67) | 0.643 | -0.03 | (-0.56,0.50) | 0.918 |

^a^ Abbreviations: OA = osteoarthritis, Tt. = total, Ct. = cortical, Tb. = trabecular, FEA = finite element analysis, Th = thickness, N = number, CI = confidence interval

^b^ Model adjustments: age, BMI, fracture since age 50, smoking (ever vs. never), drinking (≥2, 0-2, vs. 0 drinks/week), physical activity (ActivPAL4^TM^, steps/day), femoral neck BMD T-score, pain medication.

**Supplementary Table 5c Associations between standardized HR-pQCT parameters at the distal radius (DR) and persistent functional knee pain when walking along a flat surface in the SOMMA Bone and Knee OA ancillary studies as determined from multivariable linear regression models.**

|  | knee pain when walking flat (yes vs. no) | | | | | |
| --- | --- | --- | --- | --- | --- | --- |
|  | Men (N=76) | | | Women (N=111) | | |
| **DR HR-pQCT** | B | 95% CI | p-value | B | 95% CI | p-value |
| Tt. vBMD | 0.05 | (-0.88,0.98) | 0.913 | -0.30 | (-0.99,0.39) | 0.396 |
| Tt. Area | -0.88 | (-2.02,0.26) | 0.134 | 0.27 | (-0.50,1.05) | 0.490 |
| Ct. vBMD | 0.40 | (-0.69,1.49) | 0.472 | -0.04 | (-0.79,0.71) | 0.922 |
| Ct. Area | 0.18 | (-0.81,1.17) | 0.724 | -0.13 | (-0.82,0.56) | 0.707 |
| Ct. Th | 0.19 | (-0.82,1.21) | 0.712 | -0.23 | (-0.97,0.50) | 0.534 |
| Tb. vBMD | -0.35 | (-1.13,0.43) | 0.379 | -0.23 | (-0.88,0.41) | 0.481 |
| Tb. Area | -0.50 | (-1.56,0.56) | 0.358 | 0.26 | (-0.49,1.02) | 0.496 |
| Tb. Th | 0.44 | (-0.47,1.34) | 0.350 | 0.03 | (-0.67,0.74) | 0.929 |
| Tb. N | -0.80 | (-1.66,0.05) | 0.069 | -0.15 | (-0.78,0.49) | 0.654 |
| FEA failure load | 0.13 | (-0.76,1.01) | 0.776 | -0.10 | (-0.80,0.59) | 0.770 |

^a^ Abbreviations: OA = osteoarthritis, Tt. = total, Ct. = cortical, Tb. = trabecular, FEA = finite element analysis, Th = thickness, N = number, CI = confidence interval

^b^ Model adjustments: age, BMI, fracture since age 50, smoking (ever vs. never), drinking (≥2, 0-2, vs. 0 drinks/week), physical activity (ActivPAL4^TM^, steps/day), femoral neck BMD T-score, pain medication.

**Supplementary Table 6a Sensitivity analysis: Associations between standardized HR-pQCT parameters and radiographic knee OA (as determined by KLG, with levels 3-4 indicative of advanced disease) at the tibio-femoral (TF) joint in the SOMMA Bone and Knee OA ancillary studies as determined from multivariable linear regression models.**

|  | Radiographic knee OA at TF joint (KLG 3-4 vs. 0-2) | | | | | |
| --- | --- | --- | --- | --- | --- | --- |
|  | Men (N=82) | | | Women (N=121) | | |
| **DT HR-pQCT** | B | 95% CI | p-value | B | 95% CI | p-value |
| Tt. vBMD | -0.09 | (-0.63,0.44) | 0.737 | 0.14 | (-0.25,0.53) | 0.481 |
| Tt. Area | 0.06 | (-0.53,0.65) | 0.839 | -0.43 | (-0.85,-0.00) | 0.051 |
| Ct. vBMD | -0.12 | (-0.68,0.43) | 0.661 | -0.23 | (-0.62,0.17) | 0.266 |
| Ct. Area | -0.07 | (-0.57,0.42) | 0.773 | 0.13 | (-0.24,0.51) | 0.489 |
| Ct. Th | -0.06 | (-0.60,0.47) | 0.818 | 0.23 | (-0.17,0.63) | 0.258 |
| Tb. vBMD | 0.02 | (-0.46,0.49) | 0.944 | 0.06 | (-0.35,0.47) | 0.768 |
| Tb. Area | -0.07 | (-0.65,0.50) | 0.805 | -0.26 | (-0.70,0.17) | 0.236 |
| Tb. Th | 0.02 | (-0.51,0.54) | 0.949 | 0.10 | (-0.35,0.56) | 0.659 |
| Tb. N | -0.06 | (-0.61,0.50) | 0.843 | 0.16 | (-0.28,0.59) | 0.486 |
| FEA failure load | -0.04 | (-0.44,0.37) | 0.861 | -0.16 | (-0.50,0.18) | 0.365 |
| **DR HR-pQCT** | Men (N=75) | | | Women (N=108) | | |
| Tt. vBMD | 0.15 | (-0.35,0.66) | 0.550 | 0.06 | (-0.41,0.52) | 0.815 |
| Tt. Area | -0.47 | (-1.11,0.16) | 0.149 | -0.07 | (-0.59,0.45) | 0.800 |
| Ct. vBMD | 0.08 | (-0.53,0.69) | 0.808 | -0.17 | (-0.67,0.33) | 0.507 |
| Ct. Area | 0.18 | (-0.37,0.74) | 0.518 | -0.12 | (-0.57,0.34) | 0.621 |
| Ct. Th | 0.17 | (-0.39,0.73) | 0.553 | 0.00 | (-0.49,0.50) | 0.985 |
| Tb. vBMD | 0.02 | (-0.43,0.47) | 0.927 | 0.20 | (-0.23,0.63) | 0.362 |
| Tb. Area | -0.40 | (-0.97,0.17) | 0.170 | -0.02 | (-0.53,0.49) | 0.932 |
| Tb. Th | -0.20 | (-0.71,0.31) | 0.448 | 0.44 | (-0.02,0.90) | 0.062 |
| Tb. N | 0.34 | (-0.15,0.84) | 0.179 | -0.02 | (-0.45,0.40) | 0.915 |
| FEA failure load | -0.01 | (-0.51,0.49) | 0.966 | 0.01 | (-0.46,0.47) | 0.973 |

^a^ Abbreviations: OA = osteoarthritis, KLG = Kellgren Lawrence Grade, Tt. = total, Ct. = cortical, Tb. = trabecular, FEA = finite element analysis, Th = thickness, N = number, CI = confidence interval

^b^ Model adjustments: age, BMI, fracture since age 50, smoking (ever vs. never), drinking (≥2, 0-2, vs. 0 drinks/week), physical activity (ActivPAL4^TM^, steps/day), femoral neck BMD T-score, osteoporosis medication.

**Supplementary Table 6b Sensitivity analysis: Associations between standardized HR-pQCT parameters and radiographic knee OA (as determined by KLG, with levels 2-4 indicative of any disease) at the tibio-femoral (TF) joint in the SOMMA Bone and Knee OA ancillary studies as determined from multivariable linear regression models.**

|  | Radiographic knee OA at TF joint (KLG 2-4 vs. 0-1) | | | | | |
| --- | --- | --- | --- | --- | --- | --- |
|  | Men (N=82) | | | Women (N=121) | | |
| **DT HR-pQCT** | B | 95% CI | p-value | B | 95% CI | p-value |
| Tt. vBMD | -0.08 | (-0.55,0.39) | 0.735 | 0.05 | (-0.31,0.40) | 0.795 |
| Tt. Area | 0.23 | (-0.28,0.75) | 0.372 | -0.20 | (-0.60,0.19) | 0.316 |
| Ct. vBMD | 0.16 | (-0.32,0.65) | 0.507 | -0.03 | (-0.40,0.34) | 0.874 |
| Ct. Area | 0.03 | (-0.41,0.46) | 0.907 | 0.29 | (-0.05,0.64) | 0.095 |
| Ct. Th | -0.11 | (-0.58,0.36) | 0.654 | 0.28 | (-0.09,0.64) | 0.143 |
| Tb. vBMD | 0.01 | (-0.41,0.43) | 0.967 | -0.15 | (-0.53,0.23) | 0.435 |
| Tb. Area | 0.15 | (-0.35,0.65) | 0.563 | -0.15 | (-0.56,0.25) | 0.455 |
| Tb. Th | -0.03 | (-0.49,0.43) | 0.888 | -0.01 | (-0.43,0.41) | 0.955 |
| Tb. N | -0.08 | (-0.57,0.41) | 0.747 | 0.08 | (-0.33,0.48) | 0.710 |
| FEA failure load | 0.14 | (-0.21,0.50) | 0.431 | -0.09 | (-0.40,0.22) | 0.579 |
| **DR HR-pQCT** | Men (N=75) | | | Women (N=108) | | |
| Tt. vBMD | -0.03 | (-0.47,0.42) | 0.907 | -0.12 | (-0.53,0.30) | 0.583 |
| Tt. Area | -0.11 | (-0.69,0.46) | 0.696 | -0.29 | (-0.76,0.17) | 0.218 |
| Ct. vBMD | 0.13 | (-0.41,0.67) | 0.647 | 0.05 | (-0.40,0.51) | 0.815 |
| Ct. Area | -0.03 | (-0.52,0.47) | 0.917 | -0.11 | (-0.53,0.30) | 0.592 |
| Ct. Th | -0.04 | (-0.53,0.46) | 0.890 | -0.06 | (-0.51,0.38) | 0.781 |
| Tb. vBMD | -0.11 | (-0.51,0.29) | 0.596 | -0.18 | (-0.58,0.21) | 0.360 |
| Tb. Area | -0.16 | (-0.67,0.35) | 0.547 | -0.12 | (-0.58,0.35) | 0.626 |
| Tb. Th | -0.20 | (-0.65,0.25) | 0.388 | -0.07 | (-0.49,0.35) | 0.740 |
| Tb. N | 0.20 | (-0.24,0.65) | 0.367 | -0.20 | (-0.58,0.19) | 0.317 |
| FEA failure load | -0.19 | (-0.63,0.25) | 0.403 | -0.17 | (-0.59,0.25) | 0.420 |

^a^ Abbreviations: OA = osteoarthritis, KLG = Kellgren Lawrence Grade, Tt. = total, Ct. = cortical, Tb. = trabecular, FEA = finite element analysis, Th = thickness, N = number, CI = confidence interval

^b^ Model adjustments: age, BMI, fracture since age 50, smoking (ever vs. never), drinking (≥2, 0-2, vs. 0 drinks/week), physical activity (ActivPAL4^TM^, steps/day), femoral neck BMD T-score, osteoporosis medication.

**Supplementary Table 7a Sensitivity analysis: Associations between standardized HR-pQCT parameters and radiographic knee OA (as determined by KLG, with levels 3-4 indicative of advanced disease) at the patello-femoral (PF) joint in the SOMMA Bone and Knee OA ancillary studies as determined from multivariable linear regression models.**

|  | Radiographic knee OA at PF joint (KLG 3-4 vs. 0-2) | | | | | |
| --- | --- | --- | --- | --- | --- | --- |
|  | Men (N=82) | | | Women (N=121) | | |
| **DT HR-pQCT** | B | 95% CI | p-value | B | 95% CI | p-value |
| Tt. vBMD | -0.41 | (-1.10,0.27) | 0.240 | -0.40 | (-0.85,0.04) | 0.080 |
| Tt. Area | 0.69 | (-0.04,1.43) | 0.070 | 0.10 | (-0.40,0.61) | 0.691 |
| Ct. vBMD | -0.46 | (-1.17,0.24) | 0.201 | **-0.87** | **(-1.31,-0.43)** | **<0.001*** |
| Ct. Area | -0.39 | (-1.02,0.24) | 0.229 | **-0.73** | **(-1.15,-0.32)** | **0.001*** |
| Ct. Th | -0.53 | (-1.21,0.14) | 0.127 | **-0.62** | **(-1.08,-0.17)** | **0.008*** |
| Tb. vBMD | -0.09 | (-0.70,0.53) | 0.777 | 0.09 | (-0.38,0.57) | 0.699 |
| Tb. Area | 0.68 | (-0.04,1.40) | 0.070 | 0.27 | (-0.24,0.77) | 0.307 |
| Tb. Th | -0.27 | (-0.94,0.40) | 0.435 | 0.02 | (-0.51,0.55) | 0.949 |
| Tb. N | 0.24 | (-0.47,0.96) | 0.509 | -0.03 | (-0.55,0.48) | 0.898 |
| FEA failure load | -0.26 | (-0.78,0.26) | 0.330 | **-0.40** | **(-0.79,-0.01)** | **0.045** |
| **DR HR-pQCT** | Men (N=75) | | | Women (N=108) | | |
| Tt. vBMD | 0.12 | (-0.54,0.78) | 0.720 | -0.36 | (-0.89,0.18) | 0.193 |
| Tt. Area | 0.04 | (-0.81,0.88) | 0.933 | 0.05 | (-0.56,0.65) | 0.875 |
| Ct. vBMD | -0.10 | (-0.89,0.70) | 0.808 | **-0.80** | **(-1.36,-0.24)** | **0.006*** |
| Ct. Area | 0.34 | (-0.38,1.07) | 0.356 | **-0.89** | **(-1.39,-0.39)** | **0.001*** |
| Ct. Th | -0.01 | (-0.73,0.72) | 0.989 | **-0.79** | **(-1.34,-0.25)** | **0.006*** |
| Tb. vBMD | 0.32 | (-0.26,0.90) | 0.277 | 0.02 | (-0.49,0.53) | 0.939 |
| Tb. Area | 0.11 | (-0.64,0.86) | 0.775 | 0.17 | (-0.42,0.77) | 0.566 |
| Tb. Th | 0.04 | (-0.63,0.71) | 0.905 | -0.15 | (-0.69,0.39) | 0.590 |
| Tb. N | 0.39 | (-0.26,1.03) | 0.246 | 0.09 | (-0.40,0.59) | 0.706 |
| FEA failure load | 0.41 | (-0.24,1.06) | 0.219 | **-0.64** | **(-1.17,-0.11)** | **0.019*** |

^a^ Abbreviations: OA = osteoarthritis, KLG = Kellgren Lawrence Grade, Tt. = total, Ct. = cortical, Tb. = trabecular, FEA = finite element analysis, Th = thickness, N = number, CI = confidence interval

^b^ Model adjustments: age, BMI, fracture since age 50, smoking (ever vs. never), drinking (≥2, 0-2, vs. 0 drinks/week), physical activity (ActivPAL4^TM^, steps/day), femoral neck BMD T-score, osteoporosis medication.

^c^ Bold text: statistically significant (p<0.05)

^d^ * denotes p < 0.05 after accounting for multiple comparison.

**Supplementary Table 7b Sensitivity analysis: Associations between standardized HR-pQCT parameters and radiographic knee OA (as determined by KLG, with levels 2-4 indicative of any disease) at the patello-femoral (PF) joint in the SOMMA Bone and Knee OA ancillary studies as determined from multivariable linear regression models.**

|  | Radiographic knee OA at PF joint (KLG 2-4 vs. 0-1) | | | | | |
| --- | --- | --- | --- | --- | --- | --- |
|  | Men (N=82) | | | Women (N=121) | | |
| **DT HR-pQCT** | B | 95% CI | p-value | B | 95% CI | p-value |
| Tt. vBMD | **-0.66** | **(-1.11,-0.21)** | **0.006*** | 0.09 | (-0.26,0.43) | 0.622 |
| Tt. Area | **0.70** | **(0.20,1.20)** | **0.008*** | -0.11 | (-0.50,0.27) | 0.565 |
| Ct. vBMD | -0.26 | (-0.75,0.23) | 0.308 | -0.31 | (-0.66,0.04) | 0.083 |
| Ct. Area | **-0.45** | **(-0.88,-0.02)** | **0.042** | -0.16 | (-0.49,0.18) | 0.355 |
| Ct. Th | **-0.59** | **(-1.05,-0.14)** | **0.013*** | -0.14 | (-0.49,0.22) | 0.451 |
| Tb. vBMD | -0.39 | (-0.81,0.03) | 0.071 | 0.26 | (-0.10,0.62) | 0.164 |
| Tb. Area | **0.66** | **(0.17,1.15)** | **0.010*** | 0.04 | (-0.35,0.42) | 0.854 |
| Tb. Th | -0.26 | (-0.72,0.21) | 0.282 | -0.13 | (-0.53,0.28) | 0.540 |
| Tb. N | -0.24 | (-0.74,0.26) | 0.348 | 0.20 | (-0.18,0.59) | 0.303 |
| FEA failure load | -0.29 | (-0.65,0.07) | 0.113 | 0.06 | (-0.24,0.37) | 0.674 |
| **DR HR-pQCT** | Men (N=75) | | | Women (N=108) | | |
| Tt. vBMD | -0.38 | (-0.82,0.05) | 0.088 | -0.16 | (-0.55,0.23) | 0.418 |
| Tt. Area | 0.50 | (-0.06,1.05) | 0.082 | -0.00 | (-0.45,0.44) | 0.985 |
| Ct. vBMD | -0.32 | (-0.85,0.21) | 0.237 | -0.23 | (-0.65,0.19) | 0.290 |
| Ct. Area | -0.06 | (-0.55,0.43) | 0.819 | -0.24 | (-0.62,0.15) | 0.234 |
| Ct. Th | -0.40 | (-0.88,0.08) | 0.111 | -0.20 | (-0.61,0.22) | 0.355 |
| Tb. vBMD | -0.33 | (-0.71,0.06) | 0.104 | -0.03 | (-0.40,0.34) | 0.857 |
| Tb. Area | 0.42 | (-0.08,0.91) | 0.107 | 0.02 | (-0.41,0.46) | 0.921 |
| Tb. Th | -0.27 | (-0.71,0.18) | 0.246 | 0.05 | (-0.35,0.45) | 0.811 |
| Tb. N | -0.25 | (-0.69,0.19) | 0.269 | -0.05 | (-0.41,0.31) | 0.769 |
| FEA failure load | -0.19 | (-0.63,0.25) | 0.398 | -0.05 | (-0.45,0.34) | 0.795 |

^a^ Abbreviations: OA = osteoarthritis, KLG = Kellgren Lawrence Grade, Tt. = total, Ct. = cortical, Tb. = trabecular, FEA = finite element analysis, Th = thickness, N = number, CI = confidence interval

^b^ Model adjustments: age, BMI, fracture since age 50, smoking (ever vs. never), drinking (≥2, 0-2, vs. 0 drinks/week), physical activity (ActivPAL4^TM^, steps/day), femoral neck BMD T-score, osteoporosis medication.

^c^ Bold text: statistically significant (p<0.05)

^d^ * denotes p < 0.05 after accounting for multiple comparison.

**Supplementary Table 8a Sensitivity analysis: Associations between standardized HR-pQCT parameters and simultaneous radiographic knee OA (as determined by KLG, with levels 3-4 indicative of advanced disease) and BPI knee pain in the SOMMA Bone and Knee OA ancillary studies as determined from multivariable linear regression models.**

|  | Radiographic knee OA with BPI knee pain | | | | | |
| --- | --- | --- | --- | --- | --- | --- |
|  | Men (N=82) | | | Women (N=121) | | |
| **DT HR-pQCT** | B | 95% CI | p-value | B | 95% CI | p-value |
| Tt. vBMD | -0.44 | (-1.29,0.41) | 0.312 | -0.08 | (-0.57,0.41) | 0.741 |
| Tt. Area | 0.91 | (0.01,1.81) | 0.051 | 0.06 | (-0.49,0.60) | 0.840 |
| Ct. vBMD | -0.33 | (-1.22,0.56) | 0.471 | **-0.67** | **(-1.16,-0.18)** | **0.009** |
| Ct. Area | 0.14 | (-0.65,0.92) | 0.733 | -0.47 | (-0.93,-0.00) | 0.051 |
| Ct. Th | -0.20 | (-1.04,0.64) | 0.647 | -0.33 | (-0.83,0.17) | 0.201 |
| Tb. vBMD | -0.20 | (-0.97,0.57) | 0.609 | 0.32 | (-0.19,0.83) | 0.223 |
| Tb. Area | 0.77 | (-0.12,1.67) | 0.095 | 0.06 | (-0.49,0.61) | 0.833 |
| Tb. Th | 0.06 | (-0.79,0.90) | 0.893 | 0.03 | (-0.54,0.60) | 0.918 |
| Tb. N | -0.41 | (-1.31,0.48) | 0.369 | **0.62** | **(0.08,1.16)** | **0.027** |
| FEA failure load | 0.34 | (-0.31,0.99) | 0.312 | -0.30 | (-0.72,0.12) | 0.164 |
| **DR HR-pQCT** | Men (N=75) | | | Women (N=108) | | |
| Tt. vBMD | 0.62 | (-0.20,1.44) | 0.146 | -0.20 | (-0.75,0.35) | 0.484 |
| Tt. Area | -0.45 | (-1.52,0.62) | 0.417 | -0.17 | (-0.79,0.46) | 0.600 |
| Ct. vBMD | 0.42 | (-0.60,1.43) | 0.424 | -0.04 | (-0.64,0.56) | 0.888 |
| Ct. Area | **1.20** | **(0.32,2.08)** | **0.010*** | -0.41 | (-0.95,0.14) | 0.147 |
| Ct. Th | **0.94** | **(0.03,1.84)** | **0.046** | -0.22 | (-0.81,0.36) | 0.459 |
| Tb. vBMD | 0.40 | (-0.34,1.13) | 0.294 | -0.05 | (-0.57,0.47) | 0.855 |
| Tb. Area | -0.38 | (-1.34,0.58) | 0.435 | -0.06 | (-0.67,0.56) | 0.860 |
| Tb. Th | 0.21 | (-0.65,1.06) | 0.638 | -0.13 | (-0.69,0.43) | 0.643 |
| Tb. N | 0.45 | (-0.37,1.27) | 0.284 | -0.08 | (-0.59,0.42) | 0.743 |
| FEA failure load | **1.07** | **(0.28,1.86)** | **0.010*** | -0.23 | (-0.79,0.33) | 0.426 |

^a^ Abbreviations: OA = osteoarthritis, KLG = Kellgren Lawrence Grade, Tt. = total, Ct. = cortical, Tb. = trabecular, FEA = finite element analysis, Th = thickness, N = number, CI = confidence interval

^b^ Model adjustments: age, BMI, fracture since age 50, smoking (ever vs. never), drinking (≥2, 0-2, vs. 0 drinks/week), physical activity (ActivPAL4^TM^, steps/day), femoral neck BMD T-score, osteoporosis medication and pain medication.

^c^ Bold text: statistically significant (p<0.05)

^d^ * denotes p < 0.05 after accounting for multiple comparison.

**Supplementary Table 8b Sensitivity analysis: Associations between standardized HR-pQCT parameters and simultaneous radiographic knee OA (as determined by KLG, with levels 2-4 indicative of any disease) and BPI knee pain in the SOMMA Bone and Knee OA ancillary studies as determined from multivariable linear regression models.**

|  | Radiographic knee OA with BPI knee pain | | | | | |
| --- | --- | --- | --- | --- | --- | --- |
|  | Men (N=82) | | | Women (N=121) | | |
| **DT HR-pQCT** | B | 95% CI | p-value | B | 95% CI | p-value |
| Tt. vBMD | **-0.85** | **(-1.51,-0.19)** | **0.014** | -0.05 | (-0.49,0.40) | 0.843 |
| Tt. Area | 0.69 | (-0.04,1.41) | 0.069 | 0.07 | (-0.43,0.57) | 0.779 |
| Ct. vBMD | -0.61 | (-1.31,0.09) | 0.094 | -0.37 | (-0.83,0.09) | 0.116 |
| Ct. Area | -0.42 | (-1.05,0.20) | 0.187 | -0.29 | (-0.72,0.14) | 0.188 |
| Ct. Th | -0.51 | (-1.18,0.16) | 0.138 | -0.22 | (-0.68,0.25) | 0.363 |
| Tb. vBMD | -0.53 | (-1.13,0.08) | 0.095 | 0.19 | (-0.28,0.66) | 0.437 |
| Tb. Area | 0.72 | (0.01,1.44) | 0.051 | 0.05 | (-0.46,0.55) | 0.852 |
| Tb. Th | 0.02 | (-0.66,0.70) | 0.955 | -0.06 | (-0.58,0.46) | 0.812 |
| Tb. N | -0.65 | (-1.36,0.06) | 0.077 | 0.49 | (-0.01,0.99) | 0.057 |
| FEA failure load | -0.22 | (-0.75,0.31) | 0.417 | -0.18 | (-0.57,0.21) | 0.363 |
| **DR HR-pQCT** | Men (N=75) | | | Women (N=108) | | |
| Tt. vBMD | 0.02 | (-0.66,0.70) | 0.954 | -0.11 | (-0.61,0.39) | 0.667 |
| Tt. Area | 0.10 | (-0.77,0.97) | 0.815 | -0.16 | (-0.72,0.40) | 0.582 |
| Ct. vBMD | -0.11 | (-0.93,0.72) | 0.799 | 0.21 | (-0.33,0.75) | 0.448 |
| Ct. Area | 0.55 | (-0.19,1.29) | 0.148 | -0.29 | (-0.78,0.21) | 0.255 |
| Ct. Th | 0.24 | (-0.51,0.99) | 0.532 | -0.13 | (-0.66,0.40) | 0.625 |
| Tb. vBMD | -0.10 | (-0.70,0.50) | 0.751 | -0.12 | (-0.59,0.35) | 0.615 |
| Tb. Area | 0.11 | (-0.67,0.90) | 0.774 | -0.12 | (-0.67,0.44) | 0.681 |
| Tb. Th | -0.06 | (-0.75,0.64) | 0.876 | -0.21 | (-0.72,0.29) | 0.408 |
| Tb. N | -0.14 | (-0.80,0.53) | 0.687 | -0.11 | (-0.57,0.35) | 0.635 |
| FEA failure load | 0.41 | (-0.25,1.08) | 0.228 | -0.19 | (-0.69,0.32) | 0.471 |

^a^ Abbreviations: OA = osteoarthritis, KLG = Kellgren Lawrence Grade, Tt. = total, Ct. = cortical, Tb. = trabecular, FEA = finite element analysis, Th = thickness, N = number, CI = confidence interval

^b^ Model adjustments: age, BMI, fracture since age 50, smoking (ever vs. never), drinking (≥2, 0-2, vs. 0 drinks/week), physical activity (ActivPAL4^TM^, steps/day), femoral neck BMD T-score, osteoporosis medication and pain medication.

^c^ Bold text: statistically significant (p<0.05)

^d^ * denotes p < 0.05 after accounting for multiple comparison.
