# Supplementary figures and images for: Sex-Specific Associations of Radiographic Knee Osteoarthritis and Pain with Distal Tibia Bone Microarchitecture: the Study of Muscle, Mobility and Aging (SOMMA)
Source: Calcif Tissue Int. 2026 Apr 27;117(1):65. doi: 10.1007/s00223-026-01531-9 (PMC13111493; doi:10.1007/s00223-026-01531-9)

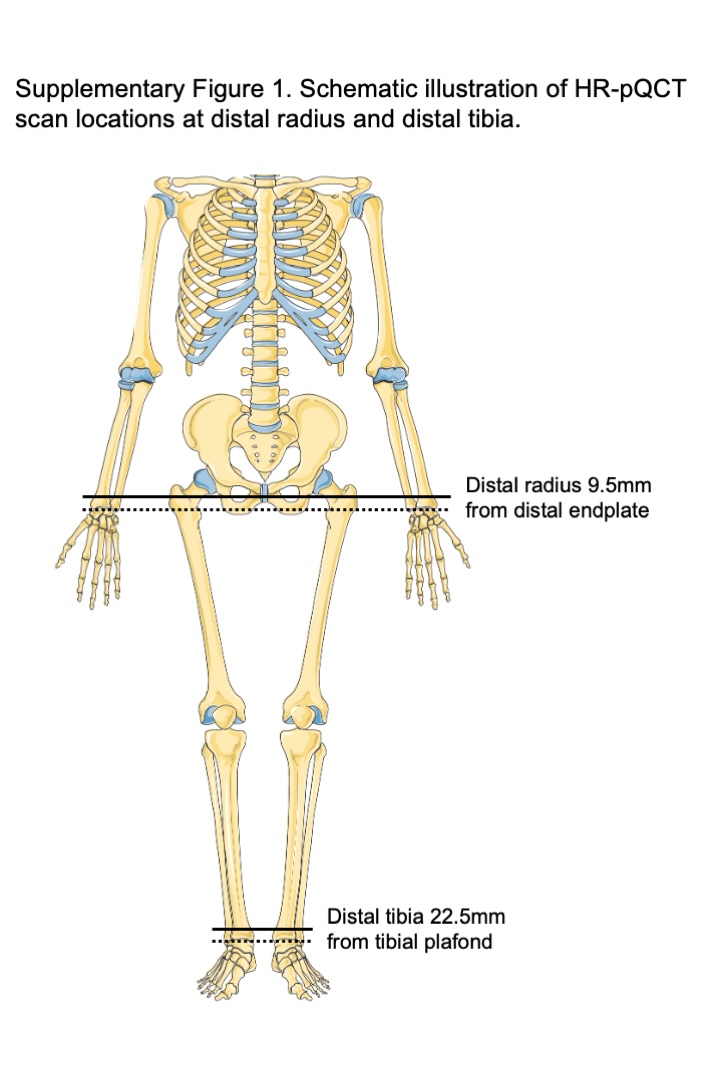

Supplement: Supplementary file 2 — Supplementary Material 2 [file 223_2026_1531_MOESM2_ESM.jpg]
